# FCRL3 is an immunoregulatory receptor that restrains the activation of human memory T lymphocytes

**DOI:** 10.1084/jem.20242474

**Published:** 2025-10-15

**Authors:** Niccolò Bianchi, Elena Foli, Mehrpouya Mostanfar, Roberta Marzi, Mara Cetty Spinella, Sara Polletti, Matteo Pecoraro, Antonino Cassotta, Roshan Thakur, David Jarrossay, Federica Sallusto, Gioacchino Natoli, Silvia Monticelli

**Affiliations:** 1 Institute for Research in Biomedicine, Università della Svizzera italiana, Bellinzona, Switzerland; 2 Department of Experimental Oncology, IEO, European Institute of Oncology IRCCS, Milan, Italy; 3 Institute for Microbiology, ETH Zurich, Zurich, Switzerland

## Abstract

Genetic variants in the *FCRL3* gene are linked to autoimmune disorders. However, the functional properties of FCRL3-expressing T lymphocytes, and the regulation and functional impact of FCRL3 expression remain understudied. Here, we performed a multiomic and functional analysis of human T lymphocytes expressing FCRL3. FCRL3 expression correlated with reduced capacity of T cells to undergo activation and was accompanied by functional specialization toward a cytotoxic phenotype, resembling cytotoxic CD4^+^ T lymphocytes and CD8^+^ effector memory T_EMRA_ cells. FCRL3 expression was induced upon repetitive TCR engagement, and sufficed to attenuate T cell responses, indicating a role as a negative regulator of the activation of differentiated T cell subsets with high cytotoxic capacity. Mechanistically, the cytoplasmic domain of FCRL3 engaged inhibitory molecules, suggesting a direct role in limiting activating signals. Overall, our study establishes FCRL3 as a functional immunoregulatory receptor that restrains the activation of highly specialized human memory T cells.

## Teaser

The authors found that the FCRL3 receptor is expressed upon repetitive activation of human memory T lymphocytes and limits their responses via inhibitory pathways. This study identifies FCRL3 as a regulator of highly differentiated memory T cells in humans.

## Graphical Abstract

**Figure:**
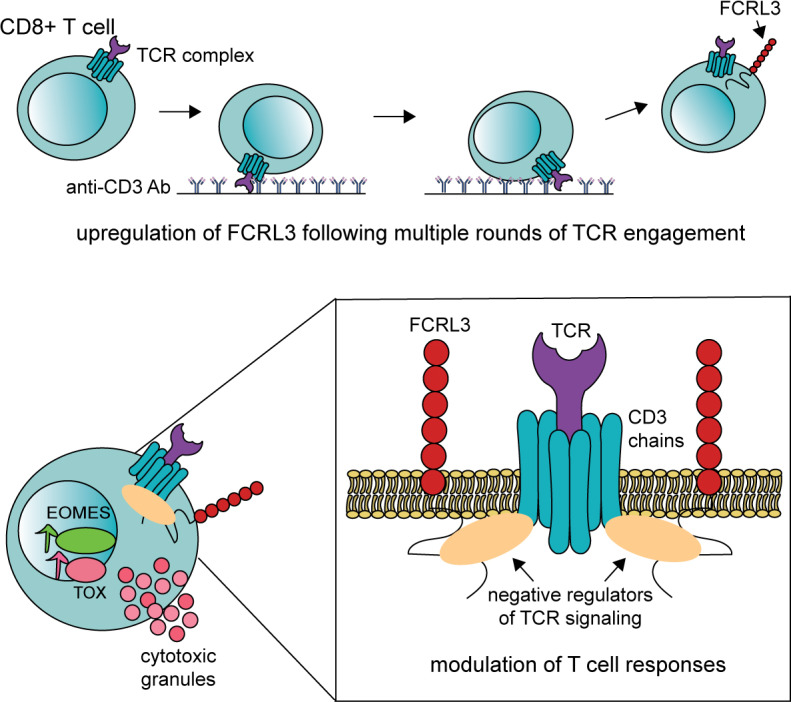

## Introduction

The dynamic interplay between costimulatory and coinhibitory receptors during T cell activation plays a critical role in shaping immune responses and in maintaining immune homeostasis. Disruption of such regulatory circuits on the one hand contributes to the pathogenesis of autoimmune diseases through the dysregulation of immune tolerance, and on the other can be harnessed to enhance cancer immunotherapy. Hence, identification of molecular players involved in such processes is essential not only to understand regulatory circuits underlying immune cell activation and regulation, but also to identify actionable therapeutic targets. Fc receptor–like protein 3 (FCRL3) is a type I transmembrane protein expressed by T and B lymphocytes and NK cells (Li et al., 2013; Schmiedel et al., 2022). It displays high homology to Fcγ receptors and contains extracellular Ig-like domains, as well as a cytoplasmic tail comprising four putative immunoreceptor tyrosine–based inhibitory motifs (ITIMs), short sequences that by recruiting inhibitory signal transducers such as protein phosphatases counteract immune cell activation (Davis, 2007; Xu et al., 2002). The immunomodulatory potential of FCRL3 is indicated by the observation that polymorphisms in the *FCRL3* gene are associated with several autoimmune disorders, including multiple sclerosis (Martínez et al., 2007; Matesanz et al., 2008), autoimmune Addison’s disease (Owen et al., 2007), and rheumatoid arthritis (Kochi et al., 2005). For instance, a specific polymorphism in the *FCRL3* promoter leads to enhanced gene expression, owing to the formation of an NF-kB binding site with increased affinity for its cognate transcription factor (Kochi et al., 2005). However, the mechanistic links between this polymorphism, lymphocyte functionality, and disease predisposition remain to be understood. Consistent with a possible regulatory role, chimeric proteins composed of murine FcγRIIB fused to human FCRL3 were shown to inhibit B cell receptor–mediated signaling in B lymphocytes, an effect that was at least in part linked to the recruitment of the phosphatase SHP1 to the FCRL3 ITIMs (Kochi et al., 2009). However, the identity of FCRL3 ligand(s) is still unknown. Indeed, although FCRL3 binds secretory IgA antibodies *in vitro* (Agarwal et al., 2020), its ability to bind antibodies *in vivo* and the potential presence of other physiological ligands for this receptor are yet to be established.

Among T lymphocytes, FCRL3 is highly expressed by a subset of Treg cells (Bin Dhuban et al., 2015; Nagata et al., 2009; Swainson et al., 2010), where it was shown to modulate cytokine production and to limit suppressive capacity (Agarwal et al., 2020). As regards conventional memory CD4^+^ and CD8^+^ T lymphocytes, we recently found that the expression of the *FCRL3* gene was increased in a subset of human effector memory T lymphocytes characterized by their limited capacity of producing inflammatory cytokines (Emming et al., 2020). However, no functional role of this transmembrane protein in T lymphocytes other than Tregs has been identified. Notably, in keeping with the extensive evolutionary divergence of FCRL family members, FCRL3 has no ortholog gene in the mouse (Davis, 2007; Li et al., 2014). The lack of animal models has insofar hampered a complete understanding of FCRL3 functions in T lymphocytes.

In this study, we aimed to determine the regulation and functional impact of FCRL3 in human T lymphocyte subsets using comprehensive molecular profiling coupled with mechanistic and functional analyses. FCRL3 expression correlated with reduced T cell activation and proliferation, as well as with increased specialization toward a cytotoxic phenotype. FCRL3 expression was induced by repetitive T cell receptor (TCR) stimulation, and the ectopic expression of the full-length receptor and even of its cytoplasmic tail was sufficient to limit T cell activation, pointing toward a direct role of FCRL3 in modulating TCR signaling. Consistent with this model, the FCRL3 protein interactome included adaptor proteins involved in the inhibition of TCR-mediated signaling. Overall, our study identifies FCRL3 as an immunoregulatory receptor induced by repetitive stimulation and capable of limiting the activation of highly differentiated memory T cells.

## Results

### FCRL3 expression modulates activation of human memory T cells

**Figure 1.**
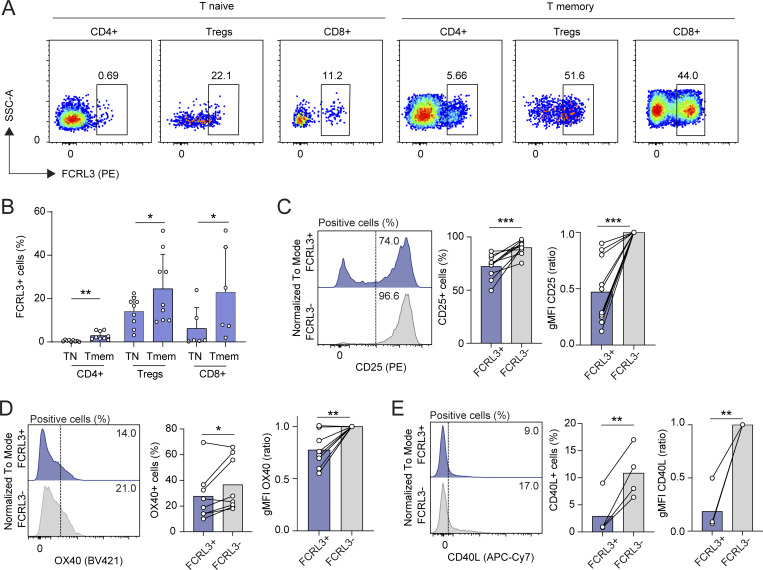
**Reduced activation of FCRL3** ^**+**^ **T cells. (A)** Surface FCRL3 expression measured by FACS in human naïve and memory CD4^+^ T helper, Tregs, and CD8^+^ T lymphocytes freshly isolated from peripheral blood of one representative healthy donor. **(B)** FCRL3 expression in different T cell populations measured as in A. Each dot represents one donor. *N* = 6–9; mean ± SD; paired *t* test, two-tailed. From left to right: **P = 0.0033, *P = 0.0303, *P = 0.0255. **(C–E)** Surface staining for CD25 (C), OX40 (D), and CD40L (E) expression in sorted FCRL3^+^ and FCRL3^−^CD8^+^ memory T cells after stimulation with plate-bound anti-CD3/CD28 for 48 h. Each dot represents a different donor, *N* = 4–10. Mean, paired *t* test or ratio paired *t* test, two-tailed. From left to right: (C)***P = 0.0008, ***P = 0.0003, (D)*P = 0.048, **P = 0.0071, (E)**P = 0.0064, **P = 0.0044. Data underlying this figure can be found in Data S4.

**Figure S1.**
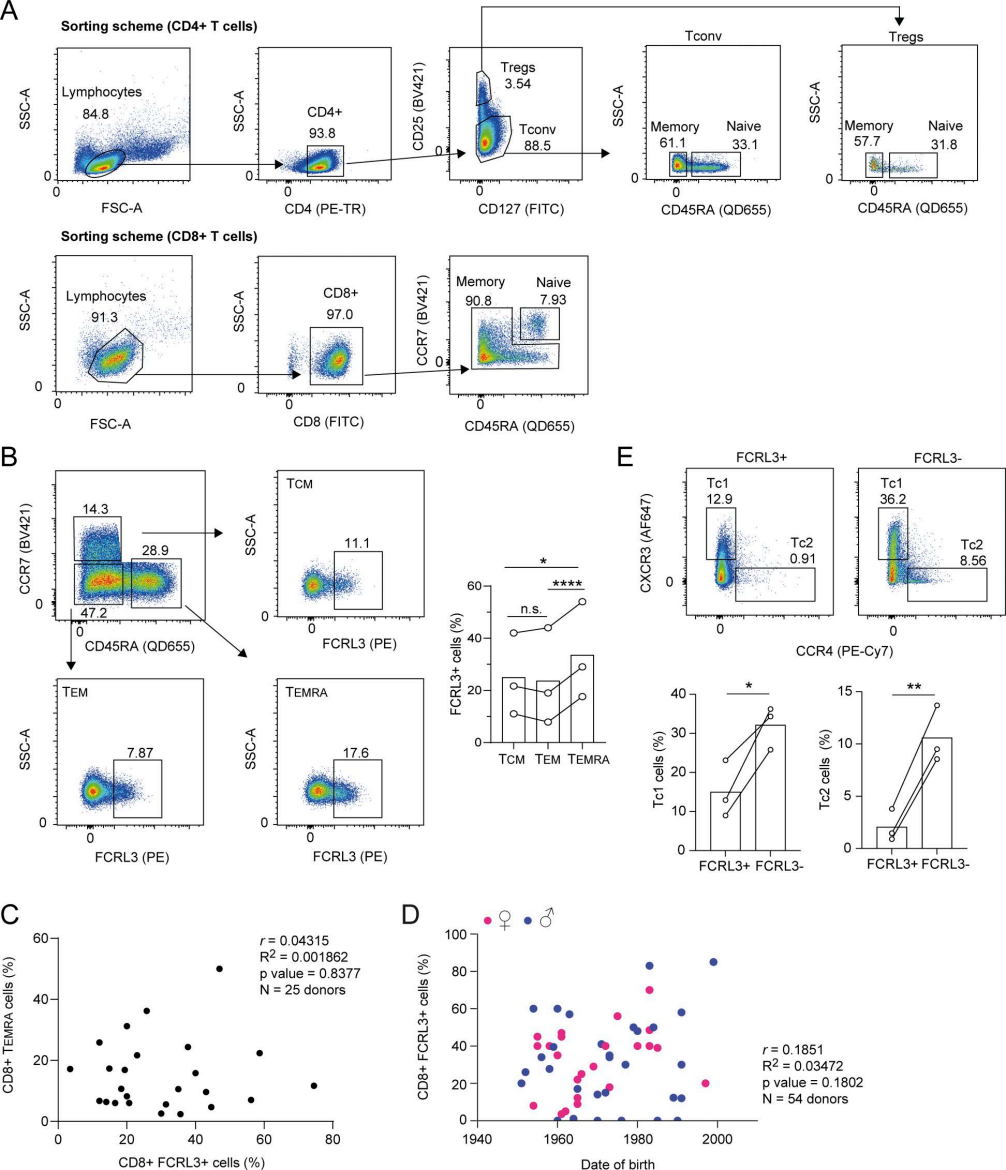
**Characterization of human FCRL3** ^**+**^ **cells. (A)** Gating strategies (sorting scheme) for the separation of human CD4^+^ T cells (conventional memory and naïve T cells and Tregs, top) and CD8^+^ lymphocytes (bottom) from peripheral blood. For conventional CD4^+^ cells, naïve T cells were defined as CD4^+^CD25^−^CD45RA^+^CCR7^+^, while memory cells were CD4^+^CD25^−^CD45RA^−^CD127^+/−^. Naïve Tregs were defined as CD4^+^CD25^hi^CD127^−^CD45RA^+^CCR7^+^, while memory Tregs were CD4^+^CD25^hi^CD127^−^CD45RA^−^CCR7^+^. Naïve CD8^+^ T lymphocytes were sorted as CD8^+^ and CD45RA/CCR7 double-positive, while memory cells were CD8^+^CD45RA^+/−^CCR7^+/−^. **(B)** FCRL3 expression in different CD8^+^ T cell subpopulations. Among the CD8^+^ memory cells CD8^+^CD45RA^+/−^CCR7^+/−^, the subpopulations were defined as T_CM_ CD8^+^CCR7^+^CD45^−^, T_EM_ CD8^+^CCR7^−^CD45^−^, and T effector memory reexpressing CD45RA (T_EMRA_) CD8^+^CCR7^−^CD45^+^. Each dot represents one donor. *N* = 3; paired *t* test, two-tailed. From top to bottom: *P = 0.0365, ****P < 0.0001, n.s. (not significant), P = 0.5185. **(C)** Pearson’s correlation coefficient between the percentage of CD8^+^ FCRL3^+^ memory T cells and the percentage of CD8^+^ T_EMRA_ cells across donors. Each dot represents one donor (*N* = 25). **(D)** Pearson’s correlation coefficient between the percentage of CD8^+^ FCRL3^+^ memory T cells and the date of birth of the donors. Each dot represents one donor (*N* = 54). **(E)** FCRL3 expression in the CD8^+^ T cell effector subsets Tc1 and Tc2. CD8^+^ memory cells CXCR3^+^CCR4^−^ were defined as Tc1, and CXCR3^−^CCR4^+^ cells were defined as Tc2. Each dot represents one donor. *N* = 3; paired *t* test, two-tailed. From left to right: *P = 0.0393, **P = 0.0066. T_CM_, T central memory; T_EM_, T effector memory. Data underlying this figure can be found in Data S4.

**Figure S2.**
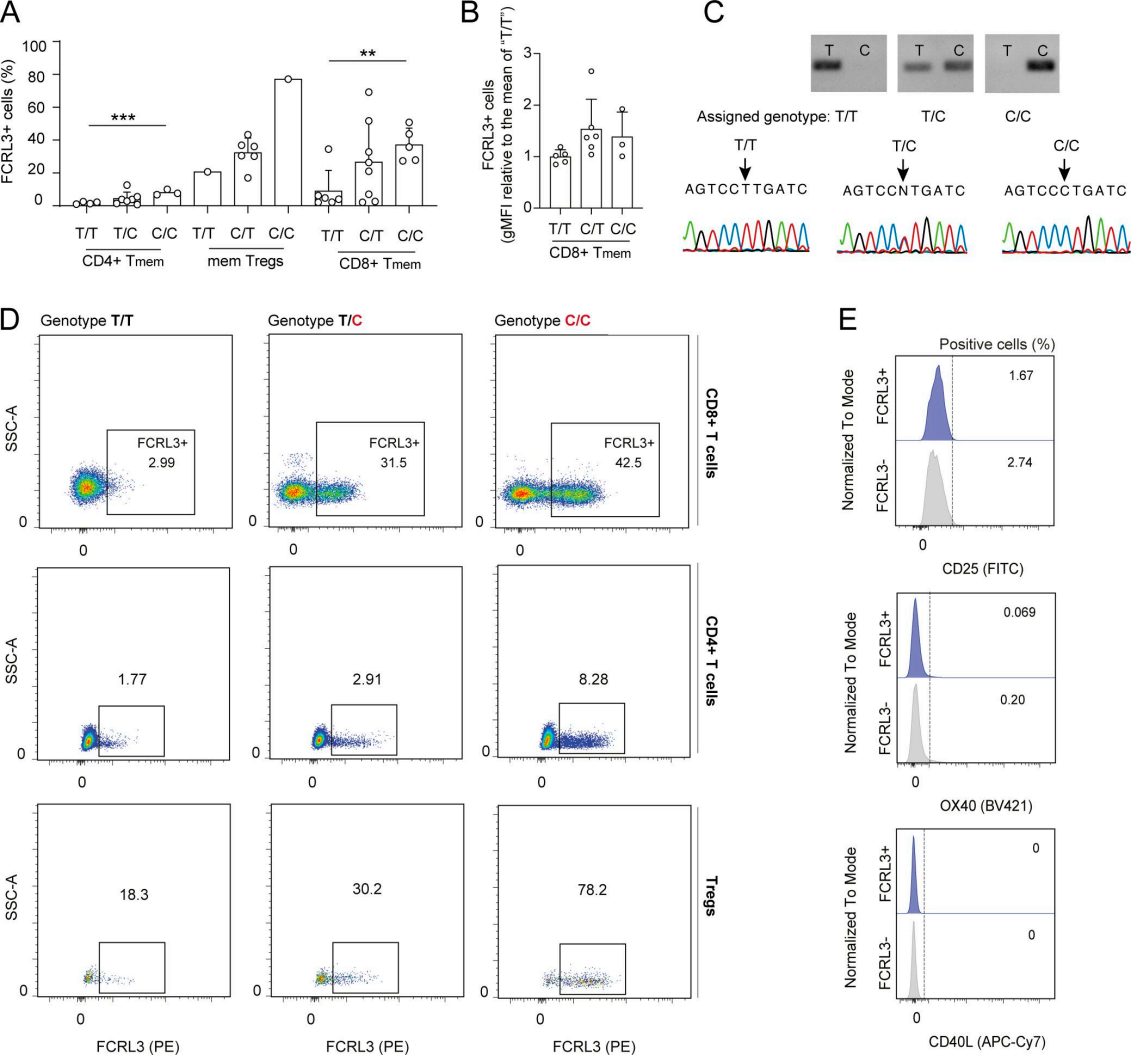
**FCRL3 expression is associated with the donors’ genotype. (A)** Percentage of surface FCRL3 expression on sorted T cell subsets from genotyped healthy donors. Each dot represents one donor. Mean ± SD; unpaired *t* test, two-tailed. ***P = 0.0009, **P = 0.003. **(B)** gMFI of gated FCRL3^+^ CD8^+^ T memory cells from genotyped healthy donors. Each dot represents one donor. Results were normalized to the average of “T/T” donors. Mean ± SD; unpaired *t* test, two-tailed. No difference was statistically significant. **(C)** Examples of genotyping results by PCR and Sanger sequencing. **(D)** Examples of FCRL3 expression in donors carrying the indicated SNP variants in the *FCRL3* promoter region (−169 bp from the transcription start site). For every donor, FCRL3 expression is shown in CD8^+^ and CD4^+^ T memory cells and Tregs. **(E)** Surface staining for the indicated AIMs (CD25, OX40, CD40L) in unstimulated CD8^+^ FCRL3^+^ and FCRL3^−^ memory T cells. gMFI, geometric mean fluorescence intensity. Data underlying this figure can be found in Data S4. Source data are available for this figure: SourceData FS2.

**Figure S3.**
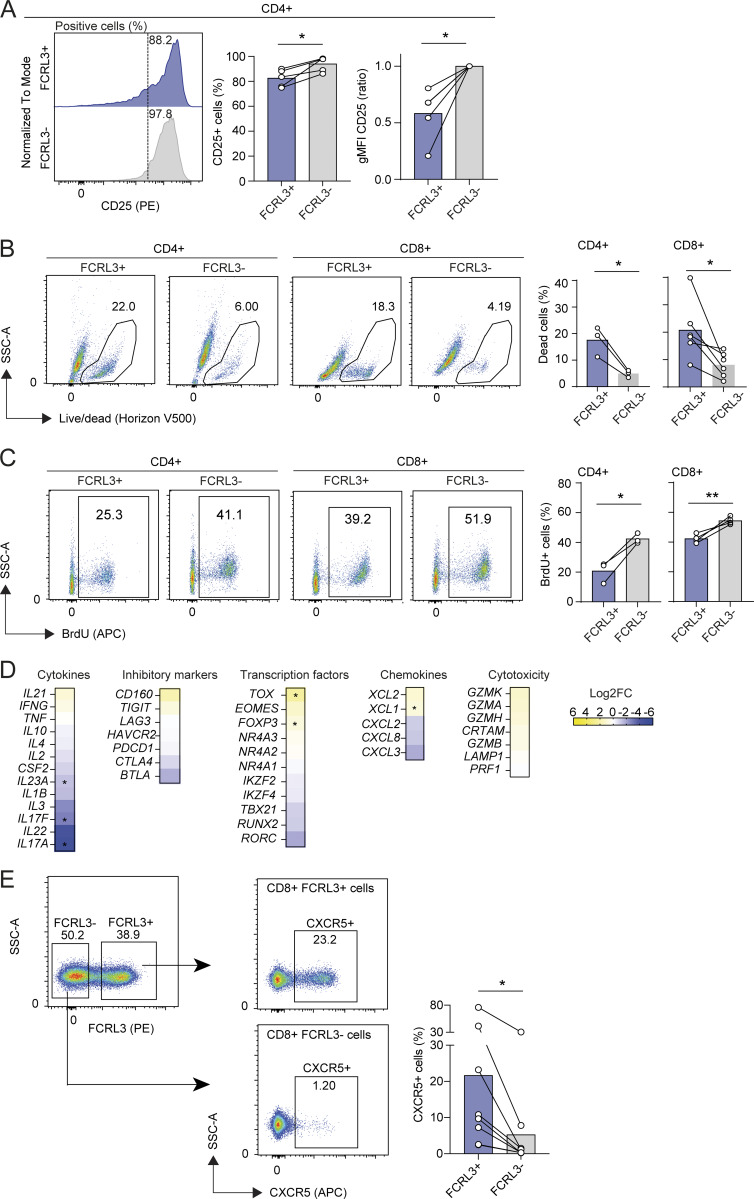
**Phenotypic characterization of FCRL3** ^**+**^ **T cells. (A)** Surface staining for CD25 expression in FCRL3^+^ and FCRL3^−^ CD4^+^ memory T cells. Cells were stimulated with plate-bound anti-CD3/CD28 for 48 h. Each dot represents one donor, *N* = 5. Mean ± SD, paired *t* test, two-tailed. From left to right: *P = 0.0174, *P = 0.015. **(B)** Live/Dead staining showing viability of sorted FCRL3^+^ and FCRL3^−^ CD4^+^ and CD8^+^ memory T cells, 3 days after activation. Each dot represents a different donor, *N* = 3–6. Mean ± SD, paired *t* test, two-tailed. From left to right: *P = 0.0369, *P = 0.0272. **(C)** BrdU incorporation assay to measure the proliferation of sorted FCRL3^+^ and FCRL3^−^ CD4^+^ and CD8^+^ memory T cells, 3 days after activation. Each dot represents a different donor, *N* = 3–4. Mean ± SD, paired *t* test, two-tailed. *P = 0.0226, **P = 0.0093. **(D)** Heatmaps showing the differential expression of selected genes from Fig. 3 A. **(E)** Surface staining for CXCR5 expression in FCRL3^+^ and FCRL3^−^ CD8^+^ memory T cells. Each dot represents one donor, *N* = 8. Mean ± SD, paired *t* test, two-tailed. *P = 0.0221.

Within the T cell compartment in the peripheral blood of healthy donors, FCRL3 was expressed by a large proportion of naïve and memory CD25^+^ Treg cells, as well as by memory CD8^+^ T cells, while expression by conventional memory CD4^+^ and naïve T cells was more limited (Fig. 1, A and B; gating strategies in Fig. S1 A). Within the CD8^+^ memory compartment, the highest expression was observed in the subpopulation of terminally differentiated CCR7^−^ CD45RA^+^ T_EMRA_ cells (T effector memory cells expressing CD45RA), compared with effector memory (CCR7^−^ CD45RA^−^, T_EM_) and central memory (CCR7^+^ CD45RA^−^, T_CM_) cells (Fig. S1 B). However, FCRL3 expression did not simply correlate with the proportion of T_EMRA_ cells in these donors (Fig. S1 C), consistent with its expression also by other subsets, including T_EM_ and T_CM_ cells (Fig. S1 B). No correlation was observed with the age and/or gender of the donors, pointing toward a general mechanism that regulates FCRL3 expression independent of sex- and aging-related processes (Fig. S1 D). Additionally, FCRL3^+^ CD8^+^ cells were not enriched for either polarized subset of Tc1 or Tc2 cells, indicating limited specialization (Fig. S1 E). FCRL3 expression was previously shown to be directly controlled by a T/C single-nucleotide polymorphism (SNP) in the *FCRL3* promoter, with the minor allelic variant (C) increasing the affinity of the transcription factor NF-kB for a cognate DNA binding motif (Gibson et al., 2009; Kochi et al., 2005; Swainson et al., 2010). Since the percentage of memory T lymphocytes expressing FCRL3 showed high donor-to-donor variability, ranging from 1 to >50% (Fig. 1 B), we hypothesized that part of this variability could be explained by allelic variation at such polymorphic site. Genotyping of the donors confirmed that the highest percentage of FCRL3 expression was indeed associated with homozygosity for the minor allele, which, however, did not significantly alter the levels of FCRL3 expression in individual cells within the FCRL3^+^ subset (Fig. S2, A–D). Consistent with their differentiated phenotype, FCRL3^+^ CD4^+^ and CD8^+^ T cells showed comparatively reduced activation in activation-induced marker (AIM) assays (Poloni et al., 2023) upon 48-h stimulation with plate-bound anti-CD3 and anti-CD28 antibodies (hereafter anti-CD3/CD28) (Fig. 1, C–E). Cells did not express these markers before activation (Fig. S2 E). Upon stimulation, the AIMs CD25, OX40, and CD40L were upregulated in both cell subsets; however, their expression was significantly attenuated in FCRL3^+^ cells (Fig. 1, C–E and Fig. S3 A), pointing toward altered activation thresholds in these cells. Additionally, FCRL3^+^ cells displayed enhanced activation-induced cell death and reduced proliferation capacities (Fig. S3, B and C), which is consistent with a differentiated T cell phenotype.

**Figure 2.**
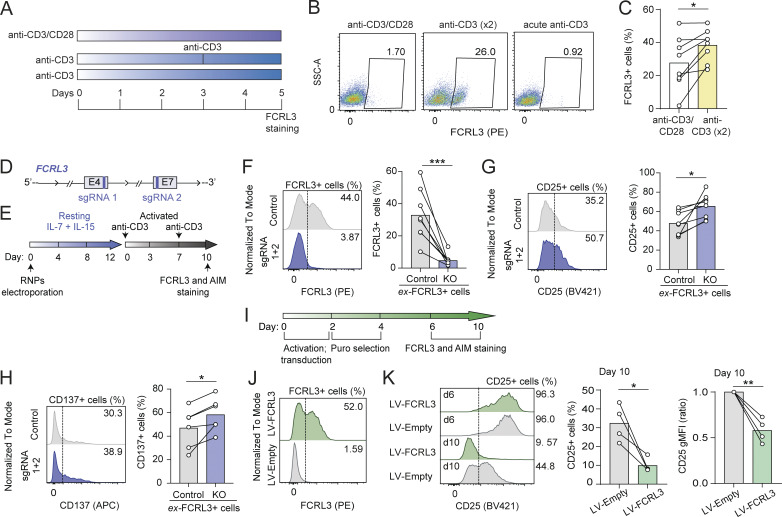
**FCRL3 expression is sufficient to modulate T cell activation. (A)** Schematic representation of the experimental setup for repetitive T cell stimulation. Memory CD8^+^ T cells isolated from peripheral blood were activated on plate-bound anti-CD3 antibody with or without costimulation with an anti-CD28 antibody or restimulation. **(B and C)** Surface expression of FCRL3 measured 5 days after activation with anti-CD3/CD28 or with two consecutive stimulations (day 0 and day 3) with anti-CD3 antibody. One representative donor (B) and *N* = 8 independent experiments (C) are shown. Each dot represents one donor. Mean; paired *t* test, two-tailed. *P = 0.0147. **(D)** Schematic representation of the *FCRL3* locus, with the exons (4 and 7) targeted by the selected sgRNAs. Only the exons targeted by the sgRNAs are shown. **(E)** Experimental workflow for FCRL3 CRISPR-KO in FCRL3^+^ CD8^+^ T cells. **(F–H)** FCRL3 (F), CD25 (G), and CD137 (H) expression on CD8^+^ FCRL3^+^ cells, transfected with RNPs containing sgRNAs targeting the *FCRL3* gene, or control. Surface expression was monitored 10 days after reactivation with anti-CD3. *N* = 6–8 independent experiments. Mean; paired *t* test, two-tailed. From left to right: (F) ***P = 0.001, (G) *P = 0.0145, (H) *P = 0.0171. **(I)** Experimental workflow for the ectopic expression of FCRL3 in CD8^+^ T cells, by lentiviral transduction. After activation with plate-bound anti-CD3/CD28 and transduction, transduced cells are selected by puromycin treatment, followed by recovery and surface staining for FCRL3 and AIMs. **(J)** Surface expression of FCRL3 in memory CD8^+^ T cells transduced with FCRL3-encoding lentivirus or empty lentivirus as a control. One representative experiment of *N* = 4. **(K)** Surface CD25 expression in FCRL3-transduced memory CD8^+^ T cells, 6 and 10 days after the initial activation. The left panel shows the result for one representative donors, while the bar graphs represent the results for *N* = 4 independent donors at day 10. The gMFI and percentage of CD25^+^ cells are both shown. Mean ± SD; paired *t* test, two-tailed. *P = 0.0306, **P = 0.0076. Data underlying this figure can be found in Data S4.

Since FCRL3-expressing cells were mostly contained within more terminally differentiated T cell compartments, we investigated whether repetitive TCR stimulation or costimulation was sufficient to induce FCRL3 expression in memory T cells. First, we selected donors with low or no expression of FCRL3, and then, we compared full stimulation with anti-CD3/CD28 with the repetitive stimulation using anti-CD3 alone. Two consecutive stimulations of memory T cells with anti-CD3 antibody were indeed sufficient to induce FCRL3 expression (Fig. 2, A–C), suggesting that FCRL3^+^ cells arise *in vivo* through repetitive encounters with recurrent or common antigens.

Since FCRL3^+^ T cells showed reduced AIM expression after TCR engagement (Fig. 1, C and D), we asked whether FCRL3 expression was causally associated with reduced T cell activation. First, we optimized an experimental system to delete FCRL3 in primary T cells by CRISPR/Cas9 and to detect the effect of its loss on T cell activation. FCRL3^+^ T cells were enriched from the peripheral blood and transfected with ribonucleoprotein (RNP) complexes containing recombinant Cas9 and sgRNAs against *FCRL3*, after which cell viability was maintained with the addition of recombinant human IL-7 and IL-15 (Fig. 2, D and E) (Albanese et al., 2022). After repetitive stimulation of these cells with anti-CD3 antibody, surface FCRL3 expression was observed on control cells, but not on cells transfected with *FCRL3* sgRNAs (Fig. 2 F). Deletion of FCRL3 was sufficient to enhance the expression of the measured AIMs (CD25, CD137), thus pointing toward a causal association between FCRL3 expression and reduced T cell activation (Fig. 2, G and H).

To confirm these findings with an orthogonal experimental approach, we ectopically expressed FCRL3 by lentiviral transduction in memory CD8^+^ T cells not expressing FCRL3 (Fig. 2 I). Since T cells needed to be preactivated for efficient lentiviral transduction and selection, in this system we could not monitor early activation events. However, we found that the ectopic expression of FCRL3 in cells preactivated with plate-bound anti-CD3/CD28 was sufficient to reduce CD25 expression at later time points (day 10) after the initial activation (Fig. 2, J and K), further underscoring the causal link between FCRL3 expression and dampened T cell activation.

Overall, FCRL3 is expressed by a subpopulation of memory T cells upon iterative TCR stimulation, and its expression is associated with reduced activation.

### Functional specialization of the FCRL3^+^ T cell subset

**Figure 3.**
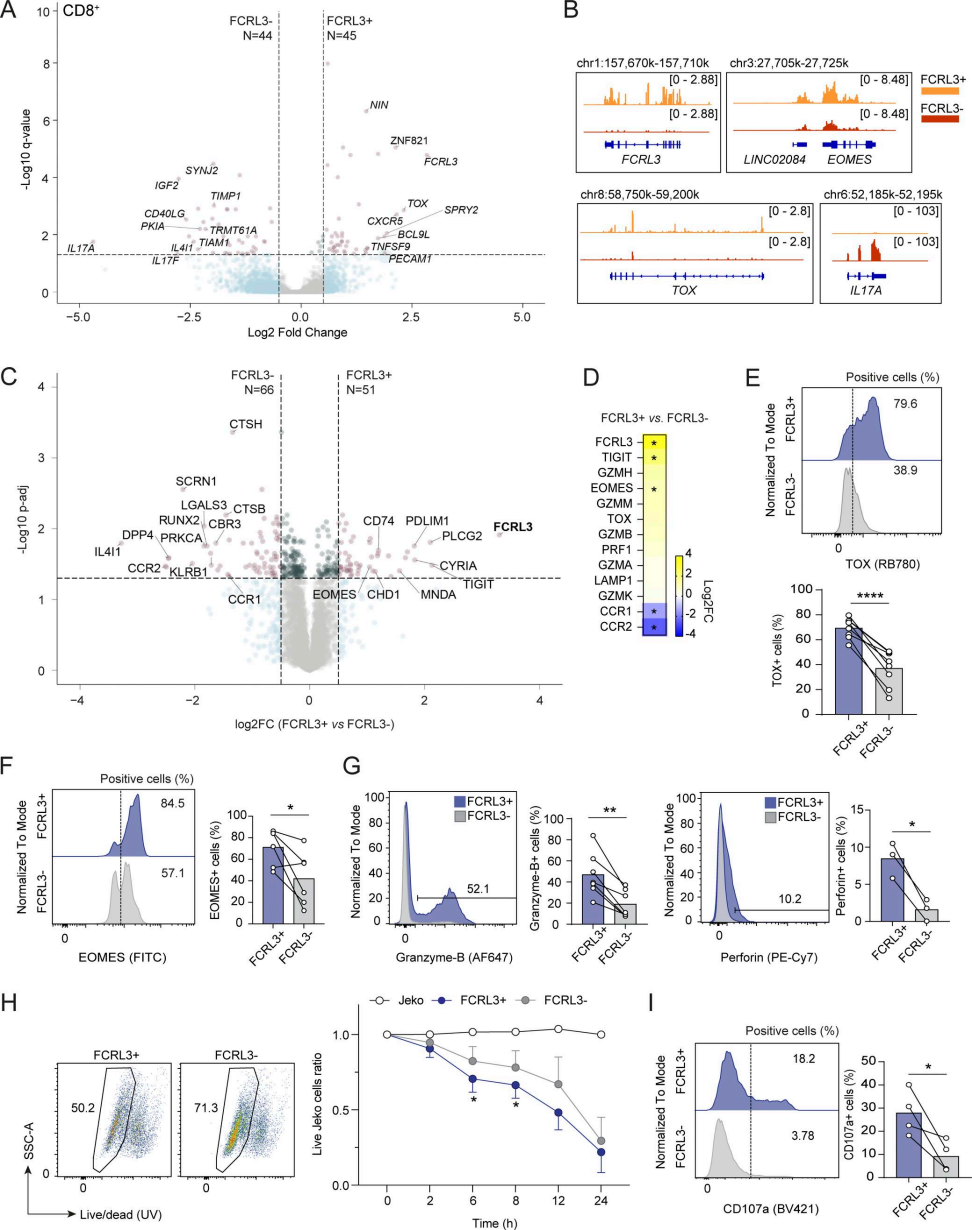
**Characterization of the CD8** ^**+**^ **FCRL3** ^**+**^ **T cell subset. (A)** Differentially expressed genes in FCRL3^+^ versus FCRL3^−^ memory CD8^+^ T cells sorted from the peripheral blood of *N* = 5 healthy donors and analyzed by RNA-seq (FDR ≤ 0.05 and log_2_FC ≥|0.5|). **(B)** RNA-seq tracks for selected transcripts enriched in FCRL3^+^ or FCRL3^−^ cells. **(C)** Differentially expressed proteins in sorted CD8^+^ FCRL3^+^ versus CD8^+^ FCRL3^−^ memory T cells, measured by shotgun mass spectrometry (log_2_FC ≥ |0.5|; FDR [Benjamini and Hochberg multiple *t* test correction] ≤0.05; *N* = 7 independent donors). **(D)** Heatmap showing the differential expression of selected proteins from C. **(E and F)** TOX (E) and EOMES (F) intracellular staining in sorted FCRL3^+^ and FCRL3^−^ memory T cells. One representative experiment and the results from *N* = 8 (TOX) or *N* = 6 (EOMES) independent donors are shown; each dot represents one donor. Paired *t* test, two-tailed. **(E)** ****P < 0.0001, (F) *P = 0.0352. **(G)** Granzyme B (left) and perforin (right) expression in sorted CD8^+^ FCRL3^+^ and CD8^+^ FCRL3^−^ memory T cells as determined by intracellular staining. The histograms show the results from different donors (*N* = 7 for granzyme and *N* = 3 for perforin). Each dot represents one donor. Mean ± SD; paired *t* test, two-tailed. From left to right: **P = 0.0018, *P = 0.0182. **(H)** Cytotoxicity of CD8^+^ FCRL3^+^ and FCRL3^−^ memory T cells. CD8^+^ FCRL3^+^ and FCRL3^−^ memory T cells were sorted and cocultured with the JeKo B cell line preincubated with the bispecific antibody blinatumomab. JeKo cell viability was then monitored with Live/Dead staining at the indicated time points. The FACS plots on the left are representative of the 8-h time point. The graph on the right shows the percentage of live JeKo cells normalized at time 0 h. *N* = 4–9 biological replicates (independent donors), mean ± SD, paired *t* test. From left to right: *P = 0.0260, *P = 0.0185. **(I)** Degranulation assay in sorted FCRL3^+^ and FCRL3^−^ memory CD8^+^ T cells. T cells were stimulated for 5 h with PMA and ionomycin, and surface CD107A (LAMP-1) expression was measured. One representative experiment and the results from *N* = 4 independent experiments are shown. Each dot represents one donor. Paired *t* test, two-tailed. *P = 0.0408. FC, fold change. Data underlying this figure can be found in Data S4.

**Figure S4.**
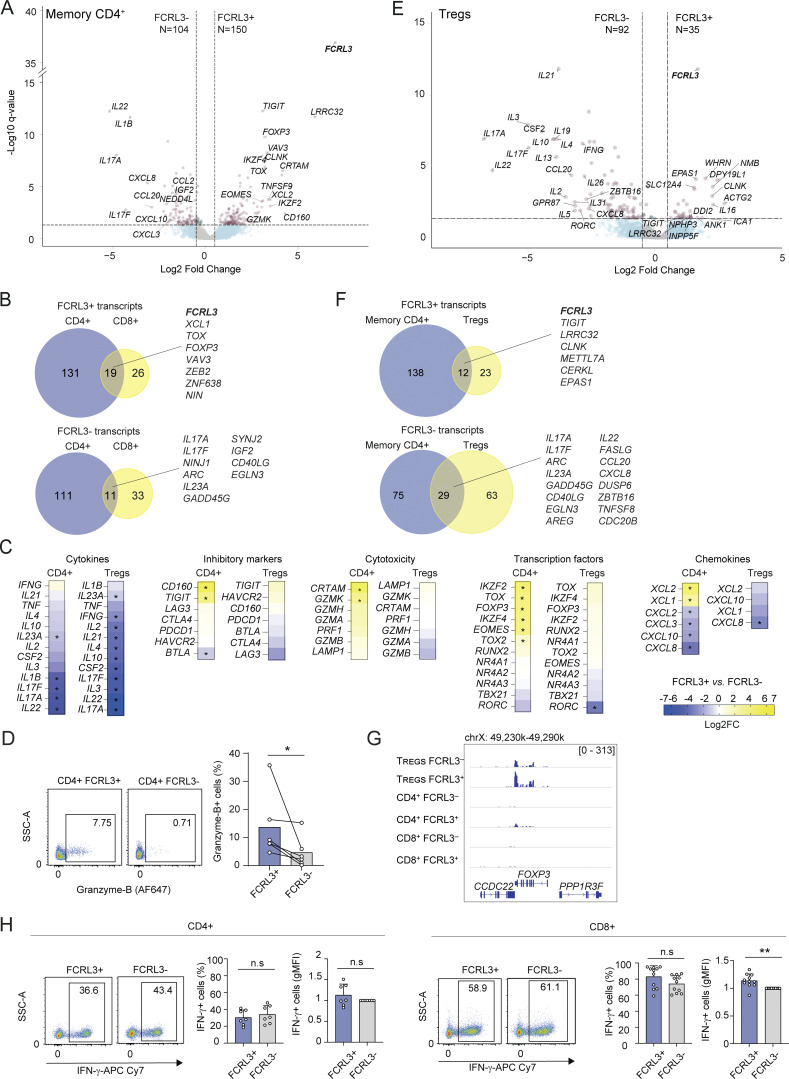
**Characterization of human CD4** ^**+**^ **FCRL3** ^**+**^ **T cells. (A)** Volcano plot showing the differentially expressed genes for FCRL3^+^ versus FCRL3^−^ memory CD4^+^ T cells from *N* = 5 independent donors, analyzed by RNA-seq (FDR ≤ 0.05 and log_2_FC ≥|0.5|). **(B)** Venn diagrams showing the intersection of the genes differentially expressed in memory CD4^+^ and CD8^+^ T cells. **(C)** Heatmaps showing the log_2_FC of selected genes from (A and E). **(D)** Granzyme B expression (by intracellular staining) in sorted CD4^+^ FCRL3^+^ and FCRL3^−^ memory T cells. *N* = 6, mean ± SD; paired *t* test, two-tailed. *P = 0.0419. **(E)** Volcano plot showing the differentially expressed genes for FCRL3^+^ versus FCRL3^−^ Treg cells from *N* = 5 independent donors, analyzed by RNA-seq (FDR ≤ 0.05 and log_2_FC ≥|0.5|). **(F)** Venn diagrams showing the intersection of the genes differentially expressed in memory conventional CD4^+^ T cells and Tregs. **(G)** Screenshot of *FOXP3* expression in FCRL3^+^ and FCRL3^−^ Tregs, conventional memory CD4^+^ and CD8^+^ T cells, by RNA-seq. **(H)** IFN-γ production by FCRL3^+^ and FCRL3^−^ CD8^+^ or CD4^+^ memory T cells. Intracellular cytokine staining was performed upon stimulation with PMA and ionomycin for 5 h. The dot plots are from one representative donor; the histograms show the results from different donors (*N* = 5–11), with each dot representing one donor; mean ± SD; paired *t* test, two-tailed. n.s.: not significant, **P = 0.004. FC, fold change. Data underlying this figure can be found in Data S4.

To further characterize the FCRL3^+^ T cell subset, we performed RNA-seq of memory CD8^+^ T lymphocytes separated from the peripheral blood of *N* = 5 healthy donors, stimulated for 3 h with phorbol 12-myristate 13-acetate (PMA) and ionomycin to induce cytokine expression. For comparison, we also performed RNA-seq of memory CD4^+^ and total Treg cells obtained from the same donors. Using a false discovery rate (FDR) ≤0.05 and log_2_ fold change ≥0.5, we found that in CD8^+^ T cells, 44 transcripts were preferentially expressed by FCRL3^−^ cells and 45 were preferentially expressed by FCRL3^+^ cells (Fig. 3, A and B; Fig. S3 D; and Data S1). Among these, *IL17A* and *IL17F* were prominently less expressed by FCRL3^+^ cells, while *TOX* and *CXCR5* were expressed at higher levels. Preferential CXCR5 expression by FCRL3^+^ cells was confirmed by surface staining, showing that indeed CXCR5^+^ cells are mostly contained within the FCRL3^+^ subset (Fig. S3 E). In CD4^+^ T lymphocytes, 104 genes had significantly reduced expression in FCRL3^+^ cells, including transcripts for inflammatory cytokines such as *IL17A*, *IL17F*, and *IL23A*, consistent with the results obtained in CD8^+^ T cells (Fig. S4, A–C; and Data S1). Interestingly, among the transcripts enriched in both CD8^+^ and CD4^+^ FCRL3^+^ cells, markers of both cytotoxicity and terminal differentiation emerged, including the transcription factors *TOX* and, to a lesser extent, Eomesodermin (*EOMES*). Increased *TOX* expression is consistent with repetitive *in vivo* TCR stimulation of these cells, since TOX is typically associated with chronic T cell stimulation (Sekine et al., 2020; Soerens et al., 2023). Specific for CD4^+^ T cells, CRTAM emerged as a marker of a cytotoxic phenotype (CD4^+^ CTLs) (Fig. S4, A–C; and Data S1). This is in agreement with the fact that EOMES directly enhances IFN-γ production, counteracts production of type-17 cytokines, and is required for the terminal differentiation of CD4^+^ CTLs, especially under conditions of chronic infection or prolonged immune activation (Geginat et al., 2023; Malyshkina et al., 2023; Pearce et al., 2003), while CRTAM is an adhesion molecule contributing to the enhancement of cytotoxic functions by CD4^+^ CTLs (Takeuchi and Saito, 2017). Consistent with this observation, we also found enhanced expression of granzyme transcripts (Fig. S4 C), and intracellular staining for granzyme B confirmed its increased expression in FCRL3^+^CD4^+^ cells (Fig. S4 D). Analysis of Treg cells from the same donors revealed reduced expression of all cytokine genes in FCRL3^+^ Treg cells, but otherwise limited overlap with the gene expression profile of conventional memory CD4^+^ T cells (Fig. S4, E and F). Although *FOXP3* emerged as a differentially expressed gene in memory CD4^+^ cells, its expression was lower than in Tregs, consistent with the observation that conventional memory human CD4^+^ T cells are able to express FOXP3 at levels that are usually insufficient to cause suppression (Sakaguchi et al., 2010) (Fig. S4 G). Indeed, both CD4^+^ and CD8^+^ FCRL3^+^ T cells maintained high expression of IFN-γ, to levels that were at least comparable to those of FCRL3^−^ cells (Fig. S4 H), suggesting reduced expression of selected cytokines but not dysfunction, and consistent with an overall CD8^+^ T_EMRA_ and CD4^+^ CTL phenotype. Our results are also concordant with single-cell RNA-seq data of tumor-infiltrating human CD4^+^ and CD8^+^ T lymphocytes, showing that in the CD4^+^ compartment, FCRL3 expression is primarily confined to Tregs and cytotoxic CD4^+^ CTLs, while within the CD8^+^ compartment, expression is more widespread but mostly localized to T_EMRA_ cells (Andreatta et al., 2022).

To further characterize the FCRL3^+^ T cell subset, we carried out shotgun proteomic analysis on FCRL3^+^ and FCRL3^−^ CD8^+^ cells obtained from *N* = 7 healthy donors. CD4^+^ T cells were not further characterized because of their lower numbers, and because their overall terminally differentiated phenotype was consistent with that of CD8^+^ T cells. By proteomic analysis, we found that 66 proteins were significantly depleted, while 51 proteins were significantly enriched in FCRL3^+^ cells (Fig. 3, C and D; and Data S2). Among the FCRL3^+^ enriched proteins, the coinhibitory receptor TIGIT and the transcription factor EOMES stood out once again for their described roles in T cell regulation. Consistent with the significant upregulation of EOMES, which especially in the context of chronic stimulation directly induces IFN-γ and granzyme expression (Geginat et al., 2023; Malyshkina et al., 2023; Pearce et al., 2003), FCRL3^+^ cells also showed enrichment of most components of the cytotoxic machinery, as well as of TOX, although below the statistical threshold (Fig. 3 D). Further analysis of TOX, EOMES, granzyme B, and perforin expression by intracellular staining of freshly isolated FCRL3^+^ CD8^+^ cells confirmed significantly increased expression of these proteins in this subset (Fig. 3, E–G).

High IFN-γ expression and granzyme expression are potentially associated with an increased ability of CD8^+^ T cells to perform efficient killing, which is also a feature of T_EMRA_ cells (Sallusto et al., 2004). We therefore investigated the killing potential of FCRL3^+^ and FCRL3^−^ CD8^+^ T cells against a target JeKo B cell line. To facilitate T cell activation and engagement with the target cells, we preincubated the JeKo cells with the bispecific T cell engager (BiTE) blinatumomab, which simultaneously binds CD19 and CD3. In pilot experiments, we tested a range of effector:target ratios and different concentrations of BiTE, and we monitored the viability of JeKo cells over time using Live/Dead staining. We then selected a concentration of BiTE (2–3 ng/ml) that led to only partial killing and a 1:1 effector:target ratio, followed by measurement of JeKo cell viability over time. We found that FCRL3^+^ cells were capable of enhanced killing compared with FCRL3^−^ T cells (Fig. 3 H), consistent with the increased expression of components of the cytolytic machinery. Similar results were obtained by staining stimulated FCRL3^+^ and FCRL3^−^ cells for the degranulation marker CD107a (LAMP-1), confirming significantly enhanced degranulation in FCRL3^+^ cells (Fig. 3 I).

Overall, our data indicate that FCRL3^+^ human T cells resemble CD8^+^ T_EMRA_ cells and acquire increased cytotoxic capacity.

### FCRL3^+^ and FCRL3^−^ cells share a common origin

**Figure 4.**
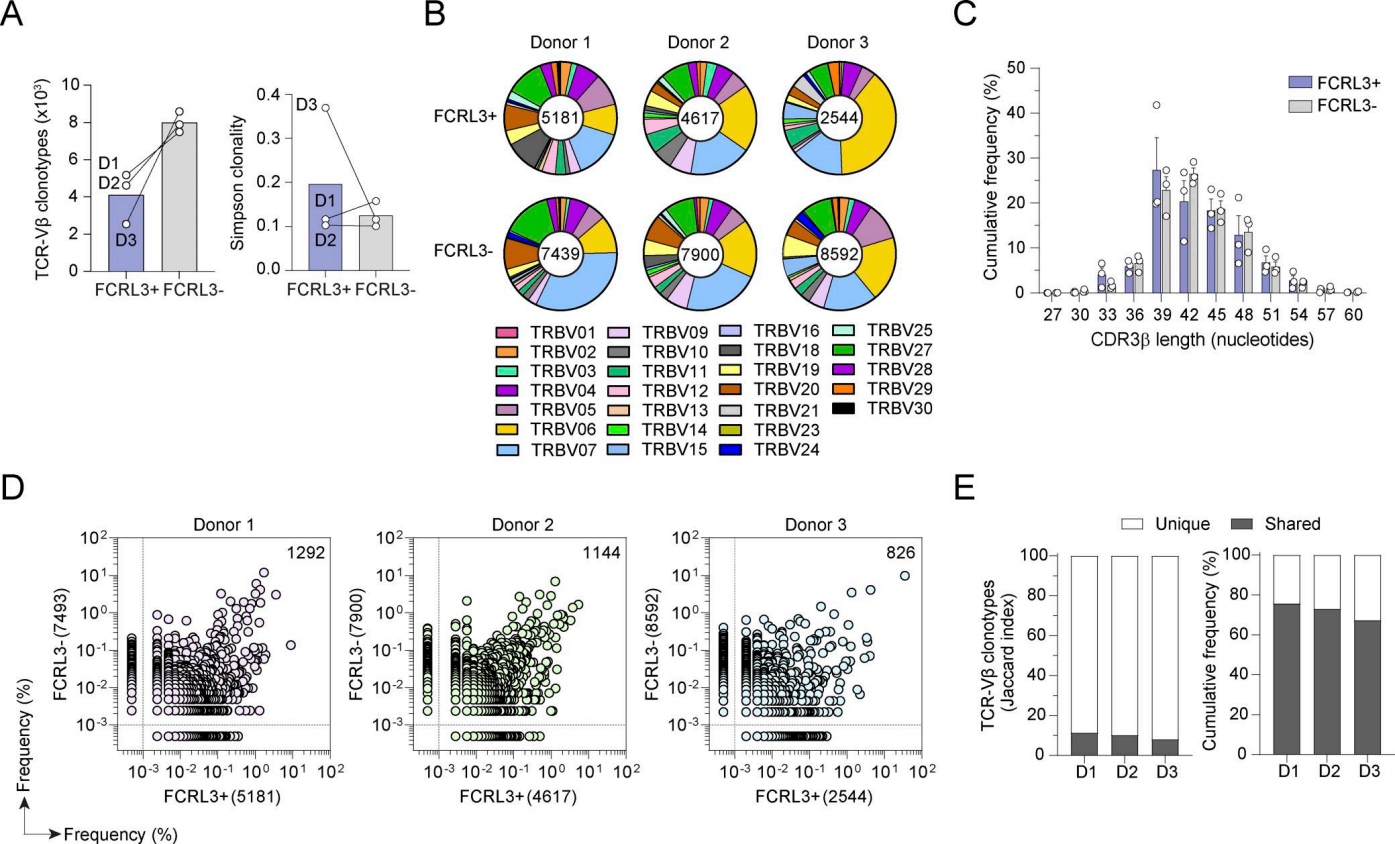
**Overlapping TCR repertoire in FCRL3** ^**+**^ **and FCRL3** ^**−**^ **cells. (A)** Number of unique productive TCR Vβ rearrangements (left) and Simpson’s clonality index (right) in sorted CD8^+^ FCRL3^+^ and CD8^+^ FCRL3^−^ memory T cell subsets from three healthy donors (D1–D3) (initial input, 5 × 10^5^ cells per subset). Each dot represents one donor (*N* = 3). Mean ± SD. **(B)** TCR Vβ gene family usage by CD8^+^ FCRL3^+^ (upper panel) and CD8^+^ FCRL3^−^ memory T cells (lower panel) in three healthy donors. Slices in the chart represent different Vβ gene families, and their size is proportional to the frequency of clonotypes using that segment. The color-coded legend is reported for the 26 different Vβ gene family. The total number of clonotypes is indicated at the center of the pie chart. **(C)** Percentage of clonotypes bearing the same CDR3β length defined by the number of nucleotides. The CDR3β length of TCR Vβ clonotypes from CD8^+^ FCRL3^+^ and CD8^+^ FCRL3^−^ memory T cells is shown in lavender and gray, respectively. Each dot represents one donor (*N* = 3). Mean ± SD. **(D)** Pairwise comparison of TCR Vβ clonotype frequency distribution in CD8^+^ FCRL3^+^ memory T cells (x axis) and CD8^+^ FCRL3^−^ memory T cells (y axis) from *N* = 3 donors. Frequencies are shown as a percentage of productive templates. Each dot indicates a unique TCR Vβ clonotype. Dots outside the dashed lines represent clonotypes that were found in only one of the two samples and that were assigned an arbitrary frequency value for graphical purposes. The total number of clonotypes is indicated in the x and y axes. Values in the upper right corner represent the number of clonotypes shared between two samples. **(E)** Unique and shared TCR Vβ clonotypes between CD8^+^ FCRL3^+^ and CD8^+^ FCRL3^−^ memory T cells in *N* = 3 donors. Shown are the percentage of clonotypes based on the Jaccard index (left) and their cumulative frequency (right). Data underlying this figure can be found in Data S4.

We found that repetitive TCR stimulation was sufficient to induce FCRL3 expression by CD8^+^ T cells, consistent with their terminally differentiated phenotype and suggesting that these T cells have undergone multiple encounters with prevalent antigens *in vivo*. To determine whether FCRL3^+^ cells derive from FCRL3^−^ cells and/or represent a clonally expanded population of cells that had undergone extensive stimulation *in vivo*, we performed TCR Vβ sequencing of CD8^+^ FCRL3^+^ and FCRL3^−^ cells from three donors. We measured thousands of productive TCR Vβ rearrangements in both subsets, spanning from 2544 to 5181 TCR Vβ clonotypes in FCRL3^+^ cells and 7493 to 8592 TCR Vβ clonotypes in the FCRL3^−^ subset. Analysis of the diversity (richness) and evenness (Simpson’s clonality) of TCR sequences showed no significant differences between FCRL3^+^ cells and FCRL3^−^ cells (Fig. 4 A). Analysis of Vβ gene family usage revealed an overall comparable distribution in the two subsets and across all three donors (Fig. 4 B), suggesting a broad repertoire without missing or overrepresented families. Furthermore, analysis of the length of CDR3β sequences showed similar distributions in both subsets (Fig. 4 C), excluding any skewing of the repertoire. To gain insights into the TCR Vβ repertoire overlap between FCRL3^+^ and FCRL3^−^ subsets, we performed pairwise comparisons of TCR Vβ frequency distribution. Within each donor, this analysis revealed a high level of clonal overlap between the two repertoires, with the most expanded clonotypes present in both FCRL3^+^ and FCRL3^−^ cells (Fig. 4 D). On average, the shared TCR Vβ clonotypes represented 10% of the total clonotypes (range 8–11%) and accounted for >70% of the sequenced templates in each subset (range 67–76%) (Fig. 4 E), indicating a substantial repertoire overlap based on reference datasets (Hu et al., 2023).

Collectively, these data indicate that FCRL3^+^ and FCRL3^−^ memory CD8^+^ T cells have rich and complete TCR Vβ repertoires that overlap extensively, suggesting a common origin followed by intraclonal diversification and FCRL3 expression.

### The intracellular portion of the FCRL3 receptor is sufficient to attenuate T cell activation

**Figure 5.**
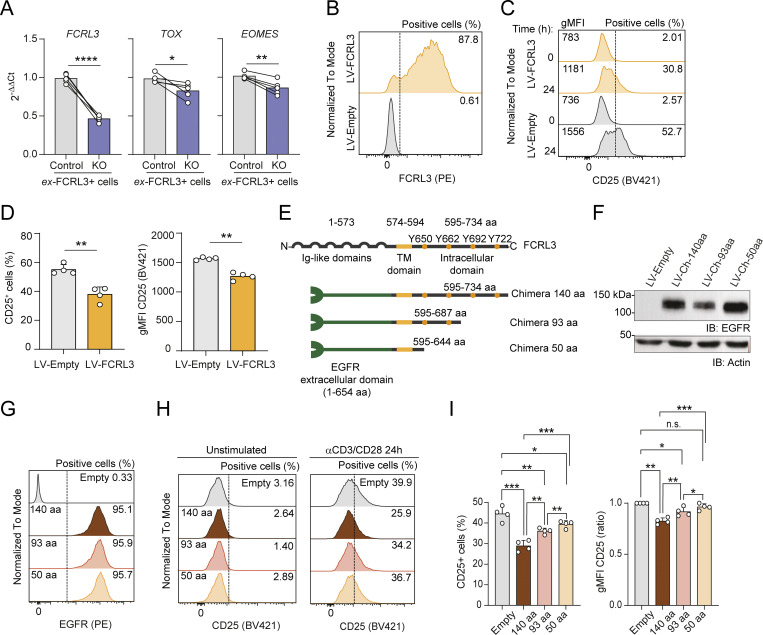
**FCRL3 expression is causally associated with reduced T cell activation. (A)** Expression of *FCRL3*, *TOX*, and *EOMES* transcripts was measured in FCRL3-KO and control cells by RT-qPCR. Transcript expression was normalized to the housekeeping gene *UBE2D2* and is shown relative to control samples. *N* = 5 independent experiments; paired *t* test, two-tailed. From left to right: ****P < 0.0001, *P = 0.0475, **P = 0.0040. **(B)** Surface expression of FCRL3 in Jurkat T cells transduced with FCRL3-lentivirus or empty lentivirus as a control. One representative experiment of *N* = 4 is shown. **(C and D)** Surface CD25 expression in FCRL3-transduced Jurkat cells. Cells were stimulated for 24 h with anti-CD3/CD28-coated beads. One representative experiment is shown in C, while the bar graphs in D show the compiled results of *N* = 4 independent experiments. The gMFI and percentage of CD25^+^ cells are both shown. Mean ± SEM; paired *t* test, two-tailed. From left to right: **P = 0.0052, **P = 0.0033. **(E)** Schematic representation of the EGFR-FCRL3 chimeric proteins. **(F)** Western blot showing the expression of the different truncated chimeric proteins in Jurkat T cells, using an anti-EGFR antibody. Data are representative of *N* = 2 experiments. **(G)** Surface expression of the EGFR in Jurkat T cells transduced to ectopically express the EGFR-FCRL3 chimeras. Data are representative of *N* = 4 independent experiments. **(H)** Surface expression of CD25 in Jurkat T cells expressing the EGFR-FCRL3 chimeras. Cells were stimulated for 24 h with anti-CD3/CD28-coated beads. One representative experiment is shown. **(I)** Surface expression of CD25 in stimulated (24 h) Jurkat T cells expressing the EGFR-FCRL3 chimeras. The compiled results of *N* = 4 independent experiments as in E are shown. Mean ± SEM; paired *t* test, two-tailed. Left panel, from top to bottom: ***P = 0.0007, *P = 0.0151, **P = 0.0046, ***P = 0.0002, **P = 0.0030, **P = 0.0013. Right panel, from top to bottom: ***P = 0.0001, n.s. (not significant), P = 0.1005, *P = 0.0326, **P = 0.0014, **P = 0.0050, *P = 0.0157. Data underlying this figure can be found in Data S4. Source data are available for this figure: SourceData F5.

Data shown above indicate that FCRL3 expression was causally associated with reduced T cell activation, since its deletion led to increased AIM expression upon TCR stimulation, while its overexpression had the opposite effect (Fig. 2). Additionally, CRISPR/Cas9-mediated deletion of FCRL3 was sufficient to reproducibly reduce the expression of two key markers of these cells, namely, the transcription factors *TOX* and *EOMES* (Fig. 5 A), raising the possibility that the modulation of TCR signaling by FCRL3 contributes to the phenotype acquired by differentiated memory T cells.

To investigate the mechanism underpinning these findings, we ectopically expressed FCRL3 in Jurkat T cells that do not express FCRL3 (Fig. 5 B). The surface expression of the TCR complex (CD3ε) and CD28 was not significantly affected by ectopic FCRL3 expression. However, upon TCR stimulation using anti-CD3/CD28-coated beads for 24 h, FCRL3^+^ Jurkat T cells showed on average a ∼31.1% reduction in CD25^+^ cells compared with control cells (Fig. 5, C and D), recapitulating the phenotype observed using primary T cells and further pointing toward an intrinsic effect of FCRL3 in the modulation of T cell activation.

To determine whether the expression of the intracellular tail of FCRL3 was sufficient to modulate TCR signaling, we generated a chimeric protein containing the intracellular and transmembrane portions of FCRL3 fused to the extracellular domain of the EGF receptor (EGFR) (Desai et al., 1993). We engineered versions of this chimera containing the full-length intracellular tail (140 amino acids, 595–734), a truncation mutant lacking about half of the C-terminal distal intracellular portion (93 aa long, 595–687), and a shorter version containing only the membrane-proximal region (50 aa, 595–644) (Fig. 5, E and F). The location and number of tyrosine residues belonging to previously identified putative ITIMs are also indicated (Kochi et al., 2009). Surface expression of these chimeric proteins in Jurkat T cells was robust and comparable across samples (Fig. 5 G). Acute stimulation with anti-CD3/CD28-coated beads led to a significant reduction in CD25 expression (∼35.0% reduction) in cells expressing the C140 full-length construct, recapitulating the effect observed with the full-length FCRL3 protein (Fig. 5, H and I). This effect on CD25 expression was gradually lost with increasing truncations of the C-terminal tail, with the largest and intermediate truncations showing minor (11.2% reduction) and intermediate (18.7% reduction) effects, respectively. This indicates that the entire length of the intracellular FCRL3 domain is required for its maximal effect.

### The FCRL3 intracellular tail interacts with negative regulators of TCR signaling

**Figure 6.**
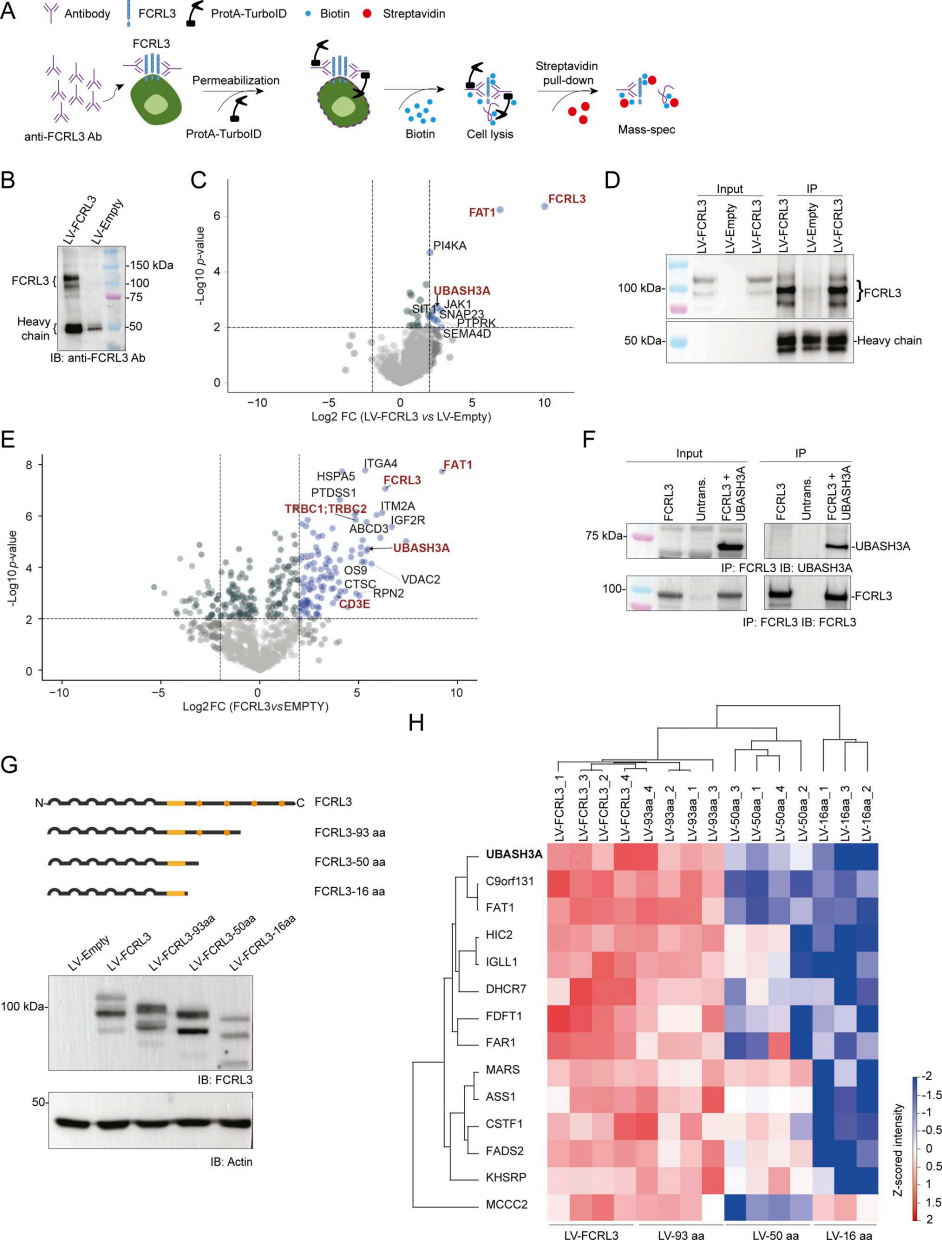
**Intracellular portion of FCRL3 interacts with UBASH3A. (A)** Schematic representation of the Protein A-TurboID experimental workflow. An anti-FCRL3 antibody was added to FCRL3-expressing or nonexpressing Jurkat cells followed by the addition of Protein A-TurboID fusion protein and biotin. After biotinylation and extensive washing, biotinylated proteins were recovered using streptavidin-conjugated beads and subjected to mass spectrometry analysis. **(B)** Example of Protein A-TurboID experiment. After incubation of FCRL3-expressing Jurkat cells (or empty vector control cells) with a mouse monoclonal anti-FCRL3 antibody together with recombinant Protein A-TurboID, biotinylated proteins were recovered by streptavidin pull-down, followed by western blot to confirm enrichment of FCRL3. The antibody used for immunoblot was a rabbit polyclonal anti-FCRL3. **(C)** Differentially retrieved proteins following FCRL3 Protein A-TurboID and mass spectrometry of FCRL3-expressing versus nonexpressing cells (log_2_FC ≥ |2|; P ≤0.01; *N* = 5 replicates). **(D)** Example of FCRL3 IP. Jurkat cells expressing FCRL3 (or empty vector control) were lysed, and 1.5 mg of protein extract was used for IP with a rabbit polyclonal anti-FCRL3 antibody, followed by western blot with the same antibody. Two independent representative FCRL3 IPs are shown. **(E)** Differentially retrieved proteins following FCRL3 IP-MS of FCRL3-expressing versus nonexpressing cells (log_2_FC ≥ |2|; P ≤0.01; *N* = 4 replicates). **(F)** Co-IP of FCRL3 with UBASH3A. HEK cells were transfected with the indicated plasmids, followed by IP of FCRL3 using a rabbit polyclonal anti-FCRL3 antibody and immunoblot for either UBASH3A (top) or FCRL3 itself (bottom). Data are representative of *N* = 2 independent experiments. **(G)** Schematic representation of the FCRL3 protein and C-terminal truncations (top). All proteins were efficiently expressed, as shown by western blot (bottom). **(H)** Heatmap showing the differentially enriched proteins by IP-MS of Jurkat cells transduced with full-length FCRL3 and its truncations. *N* = 3–4 independent samples. Significant differences between multiple experimental conditions were assessed with an ANOVA multiple-sample test (0.01 permutation-based FDR cut-off, 250 randomizations). Significant proteins were filtered, and Z-score normalization was applied to each protein across all samples. Unsupervised hierarchical clustering was employed to visualize on a heatmap proteins with a positive Z-score value for all replicates of the conditions LV-FCRL3 and LV-FCRL3-93 aa. IP, immunoprecipitation. Data underlying this figure can be found in Data S4. Source data are available for this figure: SourceData F6.

To explore the signaling molecules that may relay FCRL3 signals inside the cell, we set out to identify proteins proximal to FCRL3 in transduced Jurkat T cells. To this aim, we used a high-stringency proximity labeling approach based on the detection of FCRL3 with a specific monoclonal antibody, followed by its binding with a fusion protein consisting of protein A and TurboID, a highly efficient promiscuous biotin ligase, and the subsequent biotinylation of proteins within a 20-nm radius (Santos-Barriopedro et al., 2021) (Fig. 6, A and B). After streptavidin pull-down and mass spectrometry, we recovered FCRL3 itself and the antibody used for biotinylation, indicating successful antibody recognition and biotinylation reaction. Among the additional proteins that were significantly enriched in FCRL3-expressing samples compared with empty vector controls, we identified the transmembrane protein FAT atypical cadherin 1 (FAT1), a previously reported interactor of FCRL3 (Oughtred et al., 2021), thus further validating our dataset (Fig. 6 C). These identified proteins were not differentially expressed between FCRL3^+^ and FCRL3^−^ T cells, indicating that their proximity with FCRL3 is independent of their expression level and only depends on the expression of FCRL3 itself.

Among the biotinylated proteins proximal to FCRL3, a few prominent negative regulators of TCR signaling were recovered, including the phosphatase ubiquitin-associated and Src-homology 3 (SH3) domain containing A (UBASH3A) and SHP2-interacting adaptor protein 1, a transmembrane protein adaptor capable of limiting T cell activation by recruiting the SH2 domain–containing tyrosine phosphatase SHP2 (Marie-Cardine et al., 1999; Simeoni et al., 2005) (Fig. 6 C). In particular, UBASH3A stood out because of its high enrichment, as well as its established importance as a negative regulatory of T cell activation (Carpino et al., 2004; Ge et al., 2019; Shifrut et al., 2018), further highlighted by the identification of genetic variants in the *UBASH3A* gene that are associated with autoimmune disease, including type 1 diabetes, rheumatoid arthritis, systemic lupus erythematosus, celiac disease, and others (Smyth et al., 2008; Tsygankov, 2018). While the closely related protein UBASH3B is widely expressed, UBASH3A is highly enriched in lymphoid tissues, and specifically in T lymphocytes (Carpino et al., 2004). Both UBASH3A and UBASH3B contain an ubiquitin-associated (UBA) domain, a SH3 domain, and a phosphatase domain, which is a key functional domain capable of dephosphorylating a variety of substrates, most notably ZAP70, hence dampening TCR signaling and limiting T cell activation (Mikhailik et al., 2007; San Luis et al., 2011; Tsygankov, 2018). In addition to its direct interactions with ZAP70, UBASH3A was also reported to interact with the CD3 chains of the TCR complex, as well as with CBL and CBL-B, E3 ubiquitin ligases that act as negative regulators of T cell activation (Ge et al., 2019; Liu and Gu, 2002). The SH3 domain of UBASH3A is primarily involved in the recognition of proline-rich sequences, and this was reported to be the mode of UBASH3A recognition at least for CBL (Feshchenko et al., 2004) and CD3ε (Ge et al., 2019), while the UBA domain may interact with other target proteins depending on their ubiquitination status. Concordant with a role in suppressing T cell functions, *Ubash3a* deletion in mice led to exacerbated experimental autoimmune encephalomyelitis (Carpino et al., 2004), and deletion of the *UBASH3A* gene in primary human CD8^+^ T lymphocytes led to increased cell proliferation (Shifrut et al., 2018).

**Figure S5.**
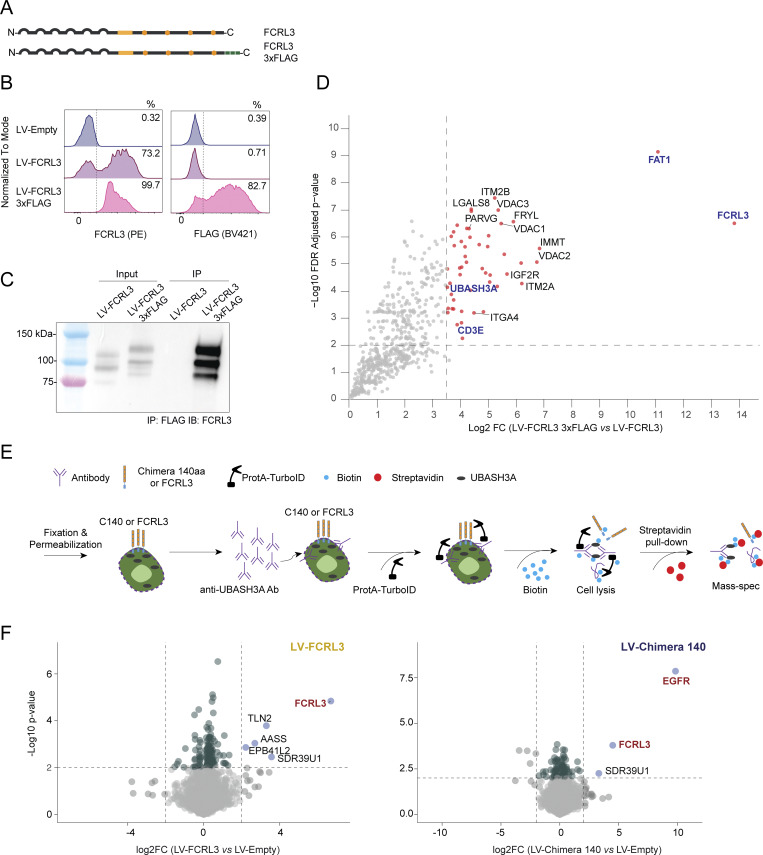
**IP-MS and TurboID-MS confirm the interaction between FCRL3 and UBASH3A. (A)** Schematic representation of FCRL3 and the C-terminally tagged FCRL3-3xFLAG protein. The 3xFLAG tag is shown as green squares. **(B)** Surface (left) FCRL3 staining and intracellular (right) anti-FLAG staining showing surface expression of the FCRL3-3xFLAG construct. **(C)** Western blot showing efficient immunoprecipitation of FCRL3-3xFLAG with anti-FLAG agarose beads in Jurkat cells expressing LV-FCRL3 and LV-FCRL3-3xFLAG. Immunoblot was performed using an anti-FCRL3 antibody. **(D)** Scatter plot showing the enriched proteins in the IP-MS of the FCRL3-3xFLAG compared with untagged FCRL3. In this experiment, to enhance the separation of true interactors from experimental noise, an additional filter was applied by selecting proteins present in at least 3 out of 4 replicates of the FCRL3-3xFLAG condition and in <2 replicates of the untagged control (FCRL3), followed by imputation of the missing values. A two-sided two-samples *t* test (0.01 permutation-based FDR cut-off, 250 randomizations) was employed to identify significant changes, and the results were visualized with a volcano plot generated with R, version 4.4.2. **(E)** Schematic representation of the Protein A-TurboID experimental workflow for UBASH3A. The anti-UBASH3A antibody was added to Jurkat cells ectopically expressing FCRL3 or the EGFR-FCRL3 140 aa chimera (or control cells), followed by the addition of Protein A-TurboID fusion protein and biotin. After streptavidin pull-down, biotinylated proteins were analyzed by mass spectrometry. **(F)** Differentially enriched proteins in the FCRL3-transduced cells versus control (left) and chimera-transduced cells versus control (right). *N* = 4 independent samples. Source data are available for this figure: SourceData FS5.

To further validate the interaction between UBASH3A and FCRL3, we used Jurkat cells ectopically expressing FCRL3 (or empty vector control cells) to immunoprecipitate FCRL3, followed by mass spectrometry (IP-MS). IP-MS of FCRL3 was efficient (Fig. 6 D), and recovered significantly enriched levels of FAT1 and UBASH3A, confirming the interaction (direct or indirect) of these proteins with FCRL3 (Fig. 6 E). Importantly, UBASH3A was retrieved using two different experimental systems based on two different antibodies targeting FCRL3 (a mouse monoclonal for proximity labeling and MS, and a rabbit polyclonal antibody for IP-MS), indicating high-confidence and robust FCRL3-UBASH3A proximity. Additional interactors involved in TCR signaling included the TCRβ chain and CD3ε. Co-IP of FCRL3 and UBASH3A was also independently confirmed in HEK cells transiently transfected with these two proteins (Fig. 6 F). A version of FCRL3 tagged with a 3xFLAG sequence at the intracellular C terminus was also generated and used for IP-MS using anti-FLAG–conjugated beads, in comparison with untagged FCRL3 (Fig. S5, A–C). Mass spectrometry of the immunoprecipitated proteins provided additional independent confirmation of the interaction of FCRL3 with UBASH3A, CD3ε, and FAT1, among other proteins (Fig. S5 D). Given the established importance of UBASH3A as a negative regulator of TCR signaling, we also set out to determine its interactome. Jurkat T cells ectopically expressing either FCRL3 or the full-length EGFR-FCRL3 C140 aa chimera (or LV-empty controls) were used for proximity biotinylation of UBASH3A, followed by mass spectrometry (schematic in Fig. S5 E). Many proteins were recovered after streptavidin pull-down and tandem MS/MS, consistent with high UBASH3A abundance in T cells and promiscuous interactions. Several interactors of UBASH3A reported in the BioGRID database were recovered, including UBASH3B, as well as components of the TCR complex (CD3ε) and regulators of TCR signaling (ZAP70, CBL) (Data S3). Importantly, FCRL3 peptides were recovered in the FCRL3- and chimera-expressing samples, while peptides corresponding to EGFR were recovered from the chimera-expressing samples, further validating the proximity of UBASH3A with the intracellular portion of FCRL3 in T cells (Fig. S5 F).

To identify the region required for the putative interaction between FCRL3 and UBASH3A, we generated truncated versions of FCRL3, containing progressively shorter intracellular regions. All proteins were efficiently expressed in Jurkat T cells (Fig. 6 G). IP-MS of full-length FCRL3 and its truncations confirmed once again the interaction between FCRL3 and UBASH3A and, additionally, revealed progressive loss of such interaction with the decreasing length of the intracellular tail, particularly in the region between aa 50 and 93 (Fig. 6 H).

Overall, these data indicate that FCRL3 expression in T cells is causally associated with reduced activation capabilities by these cells, mediated at least in part by the interaction of the FCRL3 intracellular portion with negative regulators of TCR signaling.

## Discussion

In this work, we characterized human FCRL3^+^ T lymphocytes as a subset of CD4^+^ and CD8^+^ human memory T cells characterized by a highly differentiated effector phenotype with features of cytotoxic activity. FCRL3 expression itself was induced by repetitive TCR stimulation, and its intracellular domain was sufficient to limit T cell activation. Our data indicate an immunoregulatory function for this receptor in T lymphocytes, which we suggest being mediated at least in part by its interplay with UBASH3A, a validated negative regulator of TCR signaling involved in the dephosphorylation of ZAP70 (Mikhailik et al., 2007; San Luis et al., 2011; Tsygankov, 2018). The unifying features of FCRL3^+^ cells across conventional memory CD4^+^ and CD8^+^ cells include a cytotoxic profile associated with high IFN-γ expression and reduced activation and proliferation capacity, resembling highly differentiated effector memory cells deriving from repetitive antigen stimulation. Consistent with our observations, an expanded population of human CD4^+^ CTLs characterized by perforin and granzyme expression has been identified especially in the context of chronic viral infections (Preglej and Ellmeier, 2022; Takeuchi and Saito, 2017). These cells are antigen-experienced, display a terminally differentiated functional profile (Appay et al., 2002), and were recently shown to target cytomegalovirus antigens in fibroblasts, contributing to the elimination of senescent cells in the aging human skin (Hasegawa et al., 2023). In fact, elevated levels of circulating CD4^+^ CTL cells are a feature of ultra-centenarian people (Hashimoto et al., 2019). Similarly, FCRL3^+^ cells are likely to derive from repetitive encounters with common viral/microbial antigens. The fact that FCRL3^+^ cells may derive from memory T lymphocytes undergoing multiple rounds of stimulation *in vivo* is also suggested by the increased expression of TOX, which was shown to be induced in T cells retaining effector functions even after many rounds of repetitive *in vivo* stimulation (Soerens et al., 2023). In these conditions, TOX is likely to promote cellular longevity, rather than exhaustion (Baessler and Vignali, 2024). Despite the expression of some inhibitory receptors, most notably TIGIT, FCRL3^+^ cells do not present prominent features of exhaustion, since effector functions such as IFN-γ production and killing capacity were fully maintained or even enhanced. Furthermore, the expression of other inhibitor receptors was not significantly increased. Instead, their phenotype is consistent with that of highly differentiated effector cells deriving from repetitive TCR stimulation. Consistent with this observation, we found that surface expression of FCRL3 could be induced in human T cells *in vitro* upon repetitive exposure to anti-CD3 antibody. Our data are also in line with single-cell RNA-seq data from tumor-infiltrating human CD4^+^ and CD8^+^ T lymphocytes (Andreatta et al., 2022) and with single-cell profiling of human memory CD8^+^ T cell subsets (Galletti et al., 2020). In both instances, FCRL3 expression was shown to be primarily confined to Tregs and cytotoxic CTLs in the CD4^+^ compartment, while within the CD8^+^ compartment, FCRL3 expression was more widespread to T_EM_ and T_EMRA_ lymphocytes. These observations suggest that FCRL3 expression may also in part contribute to dysfunction or exhaustion. Notably, deletion of FCRL3 reduces *TOX* expression in CD8^+^ T cell, suggesting a direct role in modulating the expression of this key transcription factor required for the exhaustion program and also implying that FCRL3 expression may negatively regulate T cell activation in tumors. However, at this stage the relative stability of FCRL3^+^ expression *in vivo* remains to be fully understood, and the possibility remains that acquisition of FCRL3 expression represents a relatively transient state for memory T cells, which can be eventually reverted upon complete removal of the stimulus.

Identifying the mechanism underlying the effect of an immunomodulatory receptor in immune cells is no trivial matter. For example, the phosphorylation of tyrosines in the ITIMs of PD-1 is followed by the recruitment of phosphatases that limit T cell proximal signaling and costimulation (Hui et al., 2017; Kamphorst et al., 2017), while the local acidification mediated by glutamic acid and proline-rich regions of LAG3 induces the dissociation of LCK from the CD4 and CD8 coreceptor molecules (Guy et al., 2022). The FCRL3 intracellular tail contains 11 prolines within the first 70 amino acids of the membrane-proximal region, but whether a similar mechanism could also be at play for FCRL3, and at least in part contribute to its effect on T cell activation, remains unknown and difficult to dissect, especially considering the unstructured nature of this part of the protein. Even well-studied receptors like PD-1 still hold surprises, since for instance it was recently shown to modulate cytoskeletal reorganization in T cells independently of its ITIMs (Paillon et al., 2023), while LAG3 was shown to require ligand-induced ubiquitination to release its cytoplasmic tail from the membrane, enabling signaling (Jiang et al., 2025). As for FCRL3, proximity labeling experiments identified several negative regulators of TCR signaling, including UBASH3A. However, these proteins are not differentially expressed by FCRL3^+^ versus FCRL3^−^ T cells, suggesting that FCRL3 expression may contribute to increasing the local concentration of negative regulators of TCR signaling. Indeed, mouse Ubash3a was shown to be part of a TCR-inducible CD6 signalosome important in the regulation of T cell activation (Mori et al., 2021).

Since the expression of the FCRL3 intracellular tail was sufficient to diminish T cell activation, it is likely that the expression of this receptor is induced concomitantly to the acquisition of functional properties by T lymphocytes that are potentially highly damaging for healthy tissues, thereby balancing immune responses. In fact, polymorphisms in the *FCRL3* gene are associated with many autoimmune diseases. As confirmed also by our data, the presence of the minor “C” allele associates with higher percentages of FCRL3 expression by T cells. However, this was true for all T cell subsets, including Tregs, in which FCRL3 was shown to affect suppressive capacity (Agarwal et al., 2020). Therefore, although induction of FCRL3 expression (and the consequent effect on T cell signaling) appears to be a normal, common process in humans, it is unclear whether the association of this gene with autoimmunity may be linked to a dysfunction of Tregs or other CD4^+^ and CD8^+^ T cell subsets, or even to other cell types on which this receptor is highly expressed, like B lymphocytes and NK cells. One additional remaining question is also linked to the ligand recognized by this receptor *in vivo*. Although FCRL3 was shown to bind sIgAs *in vitro* (Agarwal et al., 2020), at this stage it is difficult to envision a situation that might require recognition of sIgAs by a large proportion of circulating memory CD8^+^ T lymphocytes. Having firmly established the groundwork on the role of FCRL3 in human T cell subsets, the focus of future investigations will center around the possibility of identifying its physiological ligand(s) and the possibility of targeting it for therapeutic interventions.

### Limitations of this study

Although our study establishes the impact of FCRL3 expression in T cells, it remains difficult to dissect the overall impact of FCRL3 expression *in vivo* in humans, also because this receptor is expressed by additional immune cells such as NK and B cells. For instance, while FCRL3 expression is associated with a protective role in multiple sclerosis (Yu et al., 2024), the cell type predominantly responsible for this protective effect may range from Tregs, to B cells and other immune cell subsets. An additional limitation relates to the identification of the precise mechanistic details of the interaction between FCRL3 and UBASH3A. The possibility remains that some of the more labile, dynamic, or condition-specific interactions may have not been caught in our experimental systems. In particular, whether part of the negative effect of FCRL3 on signaling is also in part mediated by tyrosine phosphorylation upon T cell activation remains to be fully explored. Finally, whether FCRL3 expression represents a relatively transient state of memory T cells, or it can be “fixed” in the memory population will be the subject of future studies.

## Materials and methods

### Ethics statement

Peripheral blood from healthy donors was obtained from the Swiss Blood Donation Centers of Lugano and Basel (Switzerland), with informed consent (authorization number CE 3428 from the Comitato Etico Canton Ticino).

### Primary human T cell separation

Peripheral blood mononuclear cells (PBMCs) were separated by gradient centrifugation (Ficoll-Paque Plus; GE Healthcare), followed by positive selection of CD4^+^ or CD8^+^ T lymphocytes using magnetic beads (Miltenyi Biotec). Naïve and memory CD4^+^ T cell subsets were further separated using a FACSAria or a SORP FACSymphony S6 (BD Biosciences) as follows: naïve: CD4^+^CD25^−^CD45RA^+^CCR7^+^; total memory: CD4^+^CD25^−^CD45RA^−^CD127^+/−^; Tregs naïve: CD4^+^CD25^hi^CD127^−^CD45RA^+^CCR7^+^; Tregs memory: CD4^+^CD25^hi^CD127^−^CD45RA^−^CCR7^+/^^−^. CD8^+^ T lymphocytes were sorted as follows: naïve: CD8^+^ and CD45RA/CCR7 double-positive; memory: CD8^+^CD45RA^+/−^CCR7^+/−^. When cells were separated in the subsets FCRL3^+^ and FCRL3^−^, an anti-FCRL3 antibody (clone H5; BioLegend) was also added to the sorting. All antibodies used in this study are described in Table S1.

### T cell stimulation and culture

Acute activation of sorted cells was performed as follows: cells were stimulated for 2 days with plate-bound recombinant anti-CD3 (0.7 μg/ml; clone TR66, in-house production) (Lanzavecchia and Scheidegger, 1987) and anti-CD28 (1 μg/ml) antibodies, followed by expansion in complete medium (RPMI-1640 supplemented with 5% human serum, 1% nonessential amino acids, 1%, sodium pyruvate, 1% glutamine, penicillin, streptomycin, and 50 μM β-mercaptoethanol). In repetitive stimulation experiments, CD8^+^ memory T lymphocytes were activated with anti-CD3 antibody alone and restimulated after 3 days with anti-CD3 antibody. For experiments no longer than 5 days, cells were kept in culture without IL-2. Otherwise, recombinant human IL-2 was added at 50–100 U/ml after 3 or 5 days from activation. FCRL3 surface staining was performed after 5 or 10 days with conjugated antibody (FCRL3-PE). All antibodies and reagents used in this study are described in Table S1.

### Intracellular and surface staining

For intracellular cytokine staining, CD4^+^ T cells were stimulated for 5 h with PMA (200 nM) and ionomycin (1 μg/ml). For CD8^+^ T cells, 100 nM PMA and 0.5 μg/ml ionomycin were used. For the last 2.5 h of stimulation, brefeldin A (10 μg/ml) was added to the cells. When specified, instead of PMA and ionomycin, cells were stimulated for 6 h with plate-bound anti-CD3 and anti-CD28 antibodies, and brefeldin A (10 μg/ml) was added to the cells for the last 2 h of stimulation. After fixation (paraformaldehyde, 4%) and permeabilization (0.5% BSA and saponin in Dulbecco’s phosphate-buffered saline [DPBS]), the staining was performed with the conjugated antibodies listed in Table S1. Intracellular staining for granzyme B, perforin, TOX, and EOMES was performed on unstimulated cells, fixed, and permeabilized using eBioscience Foxp3/Transcription Factor Staining Buffer Set (Thermo Fisher Scientific) according to the manufacturer’s instructions. Staining of surface molecules was performed in MACS buffer with the conjugated antibodies listed in Table S1. Live/Dead staining was performed before fixation, after washing cells with DPBS; either Aqua Dead or Blue Dead dyes (Invitrogen) were used. To stain surface receptors (FCRL3, CD28, CD3ε), 2 × 10^5^ cells were washed once with 1X DPBS, and incubated with a primary anti-CD28 antibody (1:50) for 15 min at 4°C, followed by staining with an AF750-conjugated secondary antibody (1:300) for 30 min at 4°C. After washing, cells were stained with anti-FCRL3-PE (1:50) and anti-CD3ε-FITC (1:20) antibodies for 30 min. For FLAG detection, cells were fixed and permeabilized using IntraSure Kit (BD Biosciences), then stained intracellularly with a Brilliant Violet 421 anti-DYKDDDDK Tag antibody (1:50) for 30 min. All samples were acquired on Fortessa Flow Cytometer, FACSymphony A3 or A5 (BD Biosciences), and data were analyzed with FlowJo software.

### Cell proliferation and viability

Cell proliferation was measured using APC-BrdU Flow Kit from BD Biosciences, following the manufacturer’s instructions. After 2 days of stimulation, cells incorporated BrdU for 1 h at 37°C. Cell viability was measured using a Live/Dead staining (Invitrogen) following the manufacturer’s instructions.

### Plasmids and cloning

Plasmids were generated and modified using standard cloning techniques. To generate the pLVX-EF1α-IRES-PURO, the ZsGreen1 sequence of pLVX-EF1α-IRES-ZsGreen1 (Clontech) was substituted with a puromycin resistance gene (PuroR) from Addgene plasmid n.99636 (Montagner et al., 2016). First, AsiSI and BlpI restriction sites were added to flank the ZsGreen1 sequence of the pLVX-EF1α-IRES-ZsGreen1 by site-directed mutagenesis (QuikChange II XL Site-Directed Mutagenesis Kit, Agilent Technologies), and then used to excise the ZsGreen1 sequence. The PuroR sequence was amplified by PCR with primers containing same restriction sites and subcloned into the pLVX backbone. The *FCRL3* gene was amplified from cDNA from the Daudi cell line (CCL-213; ATCC) and then cloned into pLVX-IRES-PURO using the SpeI and NotI restriction sites. The pLVX-EF1α-EGFR-FCRL3-140 aa-chimera-puro plasmid was generated using overlap extension PCR (Higuchi et al., 1988). First, the sequence corresponding to the extracellular region of EGFR was amplified from Addgene plasmid # 11011 (Greulich et al., 2005). Then, the sequence corresponding to the transmembrane–intracellular domain of FCRL3 was amplified from pLVX-EF1α-FCRL3-IRES-puro with a forward primer containing an additional sequence complementary to the 3′ end of the newly generated extracellular EGFR amplicon. The two generated PCR products were then combined into an overlap extension PCR. In this PCR, the first 8 cycles were run without primers, allowing the added overlapping sequence of the transmembrane–intracellular domain of FCRL3 amplicon to bind pairwise to the EGFR extracellular region and start the extension, generating the full chimera. After 8 cycles, two outer primers containing the restriction sites for XbaI and NotI were added to amplify the full-length EGFR-FCRL3-140aa chimera, and the PCR was restarted for additional 27 cycles, adjusting the extension time. Finally, this amplicon was used for cloning into pLVX-EF1α-IRES-PURO using the XbaI and NotI restriction sites. For the 93 and 50 aa truncated versions of the EGFR-FCRL3 chimera, the sequences were amplified from pLVX-EF1α-EGFR-FCRL3-140 aa-chimera-puro plasmid using reverse primers containing NotI restriction sites together with the XbaI-containing forward primer used for 140 aa chimera generation. Cloning was then performed using XbaI and NotI restriction enzymes. All PCRs were performed using Platinum SuperFi II DNA Polymerase (Thermo Fisher Scientific). WT-long-UBASH3A-V5-6xHis was a gift from Patrick Concannon,  Unversity of Florida, Gainesville, FL, USA (plasmid # 192101; Addgene). To generate the versions of FCRL3 truncated in the intracellular domain, the plasmid pLVX-EF1α-FCRL3-IRES-puro served as the template for site-directed mutagenesis. Mutagenesis was performed with QuikChange II XL Site-Directed Mutagenesis Kit (Agilent) following a PCR-based approach according to the manufacturer’s guidelines. After PCR, parental methylated and hemi-methylated DNA was digested with DpnI endonuclease for 1 h at 37°C. The resulting PCR-amplified plasmid containing the mutations of interest was transformed into Stbl3-competent bacteria. Plasmid DNA was purified using E.Z.N.A. Plasmid DNA Mini Kit I (Omega Bio-Tek). To generate the FLAG-tagged version of FCRL3, a 3×FLAG epitope tag was fused in-frame to the C terminus of the FCRL3 coding sequence in the pLVX-FCRL3-puro plasmid using In-Fusion Snap Assembly Master Mix (Takara Bio) following the manufacturer’s instructions. Primer sequences used for cloning are listed in Table S1. All plasmids were first screened by restriction enzyme digestion and then verified by Sanger or next-generation sequencing (Microsynth).

### Cell transfection and T cell transduction

HEK293T cells were seeded at 8 × 10^6^ per T75 flask and cultured in DMEM supplemented with 10% FBS, penicillin, streptomycin, and 1% sodium pyruvate. After 20–24 h, HEK293T cells were transfected using polyethylenimine or Lipofectamine 3000 (Invitrogen) with the packaging vectors pMD2.G and psPAX (# 12259 and 12260; Addgene), together with a lentiviral vector encoding the gene of interest. Medium was changed 8 h after transfection. After 24–48 h, the supernatant containing lentiviral particles was filtered and PEG-8000 and NaCl were added to a final concentration of 10% and 0.3 M. After mixing at 4°C for 12–18 h, the suspension was centrifuged (1,600 × *g*, 1 h), and the pelleted lentiviral particles were resuspended in PBS. To transduce primary T cells, 1.5 × 10^5^ cells were seeded in flat-bottom 96-well plates coated with anti-CD3 anti-CD28 antibodies, and 5–10 μl of lentivirus was added to each well. 48 h after activation and transduction, cells were removed from stimuli. For vectors containing the PuroR, puromycin (2 μg/ml) was added for 48 h, followed by removal and replacement with fresh medium containing recombinant human IL-2 (50 U/ml). All experiments were performed at least 48 h after the removal of puromycin. For Jurkat cells, 5 × 10^5^ cells were plated in a 48-well plate and 5 μl of lentivirus was added to each well. Transduced cells were selected with puromycin (2 μg/ml) for 48–72 h.

### Genotyping

Genomic DNA (gDNA) was isolated from human PBMCs using the DNeasy Blood & Tissue Kit (Qiagen). PCR primers were designed to amplify the promoter region (−169 from the transcription start site) containing a T/C SNP. Primer sequences are listed in Table S1. PCR amplification was performed using KOD Hot Start DNA Polymerase (Sigma-Aldrich); 20 ng of gDNA was used as a template, and two independent PCRs were performed to amplify either the T or the minor C allele. The annealing temperature was set at 60.5°C (T allele) or 61°C (C allele).

### Reverse transcription–quantitative PCR

T cells were lysed in TRI Reagent (Molecular Research Center), and total RNA was extracted using a Direct-zol RNA MicroPrep kit (Zymo Research). cDNA was synthesized using qScript cDNA SuperMix (Quanta Bioscience). SYBR Green FastMix (Quanta Bioscience) was used to amplify target genes in QuantStudio 3 Real-Time PCR System (Thermo Fisher Scientific). For data analysis, the gene expression level between different samples was calculated using the 2^−ΔΔCT^ method; normalizing gene expression to the housekeeping gene *UBE2D2*. All primers used for RT-qPCR experiments are listed in Table S1.

### CRISPR/Cas9 gene editing

CRISPR/Cas9-mediated editing of the *FCRL3* gene was performed as described with some modifications (Emming et al., 2020; Leoni et al., 2021). Briefly, freshly isolated, resting CD8^+^ T cells were transfected with Cas9-gRNA RNPs using the Amaxa 4D-Nucleofector (Lonza). For RNP preparation, equal amounts (400 pmol) of crRNA and tracrRNA were mixed with nuclease-free duplex buffer and annealed by boiling at 95°C for 5 min followed by cooling down to room temperature. Two crRNAs targeting the *FCRL3* gene were selected, along with a nontargeting crRNA (scrambled) as a control, all obtained from Integrated DNA Technologies (IDT; sequences are listed in Table S1). Guides targeting the coding region of the *FCRL3* gene were selected based on the ON- and OFF-target scores predicted *in silico* using both the IDT-design tool and CHOP-CHOP (Labun et al., 2019). To further limit the risk of off-target effects, both *FCRL3*-targeting sgRNAs were selected to contain a minimum of four mismatches with any possible off-target genomic coding regions. The RNPs were prepared by mixing 1.5 μl of TrueCut Cas9 Protein v2 (5 μg/μl; Thermo Fisher Scientific) with 1.5 μl of annealed gRNAs. To increase transfection efficiency, polyglutamic acid (100 mg/ml; Sigma-Aldrich) was added in a ratio of gRNA:PGA 1:0.8. The mix was incubated for 20 min at room temperature. 1 × 10^6^ cells were resuspended in Lonza P3 buffer, with its supplement added at a ratio of 4.5:1, in a final volume of 22 μl. The Cas9/gRNA/PGA mixture, along with Alt-R Electroporation Enhancer (10.8 µM; IDT), was added to the transfection mix. The cell/RNP suspension was transferred to nucleofection wells, and 1 × 10^6^ T cells were electroporated using the program EH-115. Transfected cells were kept in antibiotic-free medium for 48 h; recombinant human IL-7 and IL-15 were added at 2 ng/ml for 10 min after electroporation and supplemented to the culture medium every three days to maintain cell viability. After 12 days, cells were activated with plate-bound anti-CD3 antibody (0.7 μg/ml; clone TR66, in-house production).

### RNA sequencing

Memory CD8^+^, CD4^+^ helper, and CD4^+^ Treg populations of primary human T cells obtained from *N* = 5 independent donors were sorted and separated for FCRL3 expression as described above. Following overnight incubation at 37°C in complete medium, 1–2 × 10^6^ FCRL3^+^ and FCRL3^−^ cells were stimulated for 3 h with PMA (200 nM) and ionomycin (1 μg/ml). For CD8^+^ T cells, 100 nM PMA and 0.5 μg/ml ionomycin were used. After lysis in TRI reagent (MRC), RNA extraction was performed using the Direct-Zol RNA Miniprep kit (Zymo Research). RNA-seq was carried out using the SMART-seq2 protocol (Picelli et al., 2014b) with minor modifications. Briefly, 5 ng of total RNA (RIN > 8) was reverse-transcribed with template switching using oligo(dT) primers and an LNA-containing template-switching oligo. The resulting cDNA was preamplified, purified, and tagmented with Tn5 transposase produced in-house using a described protocol (Picelli et al., 2014a). cDNA fragments generated after tagmentation were gap-repaired, enriched by PCR, and purified to create the final cDNA library. All the samples have been paired-end-sequenced on an Illumina NOVASeq 6000 platform. RNA-seq data are available at Gene Expression Omnibus (GEO) with the accession number GSE241004.

### RNA-sequencing analysis

Read quality control was performed using FastQC v0.11.9 (https://www.bioinformatics.babraham.ac.uk/projects/fastqc/). Adaptor sequences were removed using Trimmomatic v0.39 (Bolger et al., 2014). Reads were subsequently mapped to the human genome (GENCODE, version GRCh38) using HISAT2 v2.1.0 (Kim et al., 2019). HTSeq-count v2.0.2 (Anders et al., 2015) was then used to generate the table of counts containing the number of reads mapping to each feature in each sequencing sample. Differential expression analysis was performed using DESeq2 with a threshold of counts >10. For track visualization in Integrative Genomics Viewer, BigWig files were generated using the *BamCoverage* function and normalized to bins per million using the Galaxy Community (2024).

### TCR sequencing

gDNA was extracted from sorted memory CD8^+^ FCRL3^+^ and FCRL3^−^ cells from *N* = 3 independent donors using QIAamp DNA Micro Kit (Qiagen), according to the manufacturer’s instructions. gDNA quantity and purity were assessed spectrophotometrically. Sequencing of TCR Vβ CDR3 was performed by Adaptive Biotechnologies using the ImmunoSEQ assay. The assay was performed at the survey level (detection sensitivity, 1 cell in 40,000). Each clonotype was defined as a unique productively rearranged TCR Vβ DNA nucleotide sequence; data processing was done using the productive frequency of templates provided by ImmunoSEQ Analyzer V.3.0. The Simpson clonality index was used to determine the evenness of the TCR repertoire. The unique productive rearrangements were used to determine the richness of the TCR repertoire. The percentage of shared clonotypes was calculated using the Jaccard index [J = (A∩B)/(A∪B)] as the number of shared clonotypes between two subsets divided by the total number of clonotypes present in the same subset and normalized to 100.

### Cell killing and degranulation assays

After sorting CD8^+^ memory T cells based on the expression of FCRL3, cells were cocultured with JeKo cells at different ratios. Specifically, 5 × 10^4^ CD8^+^ T cells were incubated with 5 × 10^4^ JeKo cells (1:1 ratio). For the 1:5 ratio, 5 × 10^4^ JeKo cells were incubated with 1 × 10^4^ T cells, and for the ratio 1:10, 5 × 10^4^ JeKo cells were incubated with 5 × 10^3^ T cells. Prior to the coculture, JeKo cells were preincubated with the bispecific antibody against the human CD19 and CD3 antigens (InvivoGen) at a concentration of 2–3 ng/ml for 30 min at 37°C. JeKo viability was monitored with Blue Dead staining (Invitrogen) at different time points. To discriminate T cells from JeKo cells, surface staining with conjugated antibodies CD8-FITC was added. As a control for JeKo viability, JeKo cells were cultured without T cells and the experiment was performed in JeKo cell medium for optimal viability (complete RPMI with 20% FBS). All samples were acquired on a FACSymphony A5 (BD Biosciences) cytometer, and data were analyzed with FlowJo software. For CD107A (LAMP-1) degranulation assay, FCRL3^+^ and FCRL3^−^ CD8^+^ T cells were sorted and rested one night in the incubator. Cells were then activated with PMA (200 nM) and ionomycin (1 μg/ml) for 5 h. Anti-CD107A-BV421 antibody (1:400) and the protein transport inhibitor monensin (1:1,000; BD Biosciences) were also added at the same time as the stimuli. Live/Dead staining was also performed before FACS acquisition. All reagents are described in Table S1.

### Shotgun comparative proteomics

*Ex vivo*–sorted memory CD8^+^ FCRL3^+^ and FCRL3^−^ T cells (0.5 × 10^6^ cells) from *N* = 7 independent donors (all genders) were incubated overnight at 37°C. Cells were then washed twice with PBS and lysed in 8 M urea in 100 mM Tris-HCl (pH 8.0) supplemented with a cocktail of protease inhibitors (Sigma-Aldrich) and phosphatase inhibitors (PhosSTOP, Roche). Protein concentration was determined by Qubit 4 Fluorometer following the manufacturer’s instructions. Mass spectrometry was performed at the Core Facility Proteomics & Mass Spectrometry of the University of Bern (Switzerland). For Spectronaut (SN) intensity analysis, protein-level imputation was performed if there was a minimum of 2 detections in at least one group. If there were at most one nonzero values in the group for a protein, then the remaining missing values were imputed by a left-censored method (Gaussian draw). This was done on a per sample basis, drawing values from a Gaussian distribution of width 0.3× sample standard deviation centered at the sample distribution mean minus 2.5× sample standard deviation. Any remaining missing values were imputed by the maximum likelihood estimation method. Imputation was repeated 20 times. Differential expression tests were performed by applying paired Student’s *t* test on imputed SN intensities, and an associated adjusted P value (FDR-controlled Benjamini and Hochberg multiple test correction) was calculated. For downstream analysis, only the proteins detected in at least 4 out of 7 donors were used.

### Recombinant Protein A-TurboID protein purification

The plasmid encoding the recombinant Protein A-TurboID enzyme was kindly provided by Michiel Vermeulen, Radboud University, Nijmegen, The Netherlands, and the recombinant protein was expressed and purified as previously described with few modifications (Santos-Barriopedro et al., 2021). Briefly, the plasmid was transformed into *Escherichia coli* strain C3013 and cultured on LB agar supplemented with 50 μg/ml kanamycin. After overnight incubation, six colonies were selected and inoculated into 50 ml LB medium with 50 μg/ml kanamycin, forming the starter culture. For large-scale purification, 4 Liters of LB was inoculated with 40 ml of the starter culture. Cultures were grown at 37°C until reaching an OD_600_ of 0.6, followed by the addition of 2 mM isopropyl 1-thio-β-d-galactopyranoside (Sigma-Aldrich). After an additional 3 h of growth, cells were harvested by centrifugation at 4,600 × *g* for 10 min at 4°C. The resulting cell pellet was resuspended in 160 ml lysis buffer (20 mM HEPES, pH 8.0, 2 mM dithiothreitol (DTT), 500 mM NaCl, 1 mM EDTA, 10% glycerol, 0.1% NP-40, 1 mM PMSF, 1× protease inhibitor cocktail, and 10 mM imidazole). The cell suspension underwent sonication (6 cycles at 70% amplitude, 40 s ON, and 2 min OFF) and was clarified by centrifugation at 29,000 × *g* for 1 h at 4°C. The clarified lysate passed through Pierce Disposable Column (10 ml; Thermo Fisher Scientific) containing 4 ml of Ni-NTA agarose beads (Qiagen) via gravity flow. The column was washed with 30 ml of lysis buffer and then twice with 30 ml of wash buffer (10 mM Tris, pH 7.5, 500 mM NaCl, 500 μM EDTA, and 20 mM imidazole). The bound protein was eluted in 1 ml fractions with 20 ml of elution buffer 1 (50 mM Tris-HCl, pH 7.5, 300 mM NaCl, and 100 mM imidazole) and 10 ml of elution buffer 2 (50 mM Tris-HCl, pH 7.5, 300 mM NaCl, and 200 mM imidazole). Aliquots of the collected fractions were loaded onto an SDS-PAGE gel and stained with Imperial Protein Staining (Thermo Fisher Scientific) following the manufacturer’s instructions to determine the amount and purity of the enzyme. Fractions containing Protein A-TurboID protein were pooled and concentrated using a 10-kDa MWCO spin concentrator (Amicon Ultra-15, Millipore) and dialyzed overnight in storage buffer (20 mM HEPES, pH 8.0, 1 mM DTT, 150 mM NaCl, 0.1 mM EDTA). The purified protein was filter-sterilized using a 0.22-μm Millex-GP filter (Millipore) and stored in aliquots at −80°C.

### Proximity labeling and mass spectrometry

FCRL3-expressing and control cells (20 × 10^6^ cells for each sample) were stained with a mouse monoclonal anti-FCRL3 antibody (1:50, Ultra-LEAF Purified IgG2b; BioLegend) for 15 min in PBS. After removing the unbound antibody through washing with wash buffer (20 mM HEPES, pH 7.5, 150 mM NaCl, 0.5 mM spermidine, 1× proteinase inhibitor cocktail [Sigma-Aldrich], and 1× phosphatase inhibitors [Sigma-Aldrich]), Jurkat cells were treated with digitonin buffer (0.04% digitonin in wash buffer) for 10–15 min using a rotator, and permeabilization of >80% of the cells was verified using trypan blue staining. The permeabilized cells were then incubated with 4 µg of Protein A-TurboID ligase per sample in digitonin buffer, for 45 min at 4°C using a rotator. After three washes with digitonin buffer, cells were resuspended in biotinylation reaction buffer (5 mM MgCl_2_, 20 μM biotin, 1 mM ATP in digitonin buffer) and incubated for 30 min by gentle shaking at room temperature. The cells were then pelleted at 500 × *g* for 15 min at room temperature, lysed in 200 μl RIPA buffer (50 mM Tris, pH 7.8, 150 mM NaCl, 0.5% sodium deoxycholate, 0.1% SDS, 1% NP-40), and sonicated in cycles of 15 s on/10 s off in an NGS Bioruptor sonicator until the samples became almost transparent. The samples were then centrifuged at 4°C for 10 min at maximum speed to clear the lysate. The cleared lysates were incubated with 30 μl of streptavidin beads (Sigma-Aldrich) overnight, and biotinylated proteins were pulled down at 1,500 × *g* at 4°C for 2 min. Subsequently, the beads were washed four times, including two washes with RIPA buffer and two washes with PBS, to remove residual contaminants such as detergent. A similar protocol was used for UBASH3A proximity labeling, except that after washing once with wash buffer, the cells were fixed with reagent A (BD IntraSure Kit) for 5 min at room temperature, before permeabilization in digitonin buffer. The UBASH3A protein was targeted by incubating the cells with 4 μg of UBASH3A antibody (Proteintech) for 35 min in digitonin buffer. After removal of the unbound antibody with two washes in digitonin buffer, cells were pelleted at 500 × *g* for 15 min at room temperature and incubated with digitonin buffer containing 4 µg of Protein A-TurboID ligase per sample for 45 min at 4°C using a rotator. Following this step, cells underwent two washes using digitonin buffer, pelleting down at 500 × *g* for 15 min at room temperature after each washing cycle. After the second wash, cells were resuspended in the biotinylation reaction buffer, followed by lysis and sonication as described above. All the buffers used in this protocol were supplemented with 1× proteinase inhibitor cocktail (Sigma-Aldrich) and 1× phosphatase inhibitor (Sigma-Aldrich).

Sample preparation for mass spectrometry was performed exactly as described previously (Bataclan et al., 2024). Briefly, for on-bead digestion of pulled-down proteins beads were resuspended in 8 M urea, 50 mM ammonium bicarbonate buffer, followed by a reduction reaction with 10 mM DTT for 60 min at 37°C and alkylation with 50 mM iodoacetamide for 30 min at room temperature. Digestion was performed with 1 µg of Lys-C (FUJIFILM Wako Chemicals) in 8 M urea, 50 mM ammonium bicarbonate buffer for 2 h at 37°C, followed by dilution to final 2 M urea with 50 mM ammonium bicarbonate, addition of 1 µg of trypsin (Promega), and overnight digestion at 37°C, with shaking. After addition of acetonitrile to 2% and trifluoroacetic acid to 0.3% to stop the digestion reaction, digested peptides were purified with C18 StageTips (Rappsilber et al., 2007), and eluted with 80% acetonitrile, 0.5% acetic acid. The elution buffer was eliminated by vacuum centrifugation, and the purified peptides were resolved in 2% acetonitrile, 0.5% acetic acid, 0.1% trifluoroacetic acid for single-shot LC-MS/MS measurements.

### Co-immunoprecipitation and mass spectrometry

Jurkat cells either ectopically expressing FCRL3 or an empty control vector were used. After washing with 1X PBS, cells were lysed in lysis buffer (25 mM Tris-HCl, pH 8.0, 250 mM NaCl, 0.2% NP-40, 0.5 mM EDTA, 0.5 mM EGTA), supplemented with 1× proteinase inhibitor cocktail (Sigma-Aldrich) and 1X phosphatase inhibitor (Sigma-Aldrich) for 30 min on ice. The lysate was then cleared through centrifugation at 4°C at 13,000 × *g*. Protein concentration was determined with a BCA assay (Thermo Fisher Scientific, Pierce BCA Protein Assay Kit), and 2 mg of proteins was used for immunoprecipitation. To minimize nonspecific binding, the extracted protein was initially incubated with 10 μl of protein A magnetic beads (Thermo Fisher Scientific) for 1 h at 4°C using a rotator. Subsequently, the protein extract was incubated with 50 μl of protein A magnetic beads (previously washed with lysis buffer) and 2.1 µg of anti-FCRL3 antibody (HPA048022-100UL; Sigma-Aldrich) overnight at 4°C in a total volume of 400 μl. The beads were then separated using a magnetic rack and washed twice with lysis buffer and twice with 1X PBS supplemented with 1× proteinase inhibitor cocktail (Sigma-Aldrich) and 1× phosphatase inhibitor (Sigma-Aldrich). For immunoprecipitation using anti-FLAG agarose beads, Jurkat cells ectopically expressing FCRL3 or FCRL3-3×FLAG were lysed in buffer containing 25 mM Tris-HCl (pH 8.0), 250 mM NaCl, 0.2% NP-40, 0.5 mM EDTA, and 0.5 mM EGTA, supplemented with 1× protease inhibitor cocktail (Sigma-Aldrich) and 1× phosphatase inhibitor (Sigma-Aldrich). A total of 25 μl of anti-FLAG agarose beads (ChromoTek) was added to 2 mg of total cell lysate and incubated overnight at 4°C in a final volume of 500 μl. After incubation, beads were washed twice with lysis buffer and twice with 1× DPBS. The immunoprecipitated material was then directly subjected to in-solution protein digestion for mass spectrometry analysis. Sample preparation for mass spectrometry was performed exactly as described above (Bataclan et al., 2024).

### Tandem mass spectrometry and data analysis

LC-MS/MS and data analyses were performed exactly as described previously (Bataclan et al., 2024). Briefly, peptides were separated on an EASY-nLC 1200 HPLC system (Thermo Fisher Scientific) coupled online via a nanoelectrospray source (Thermo Fisher Scientific) to a Q Exactive HF mass spectrometer (Thermo Fisher Scientific). MS/MS spectra were acquired with a resolution of 15,000 at 200 m/z, maximum injection time of 55 ms, and AGC target of 1e5. Dynamic exclusion was set to 30 s to avoid repeated sequencing. The mass spectrometry raw files were processed using MaxQuant software v.1.6.7.0 (Cox and Mann, 2008). Peptides and proteins were identified with a 0.01 FDR using the integrated Andromeda search engine (Cox et al., 2011) to search spectra against the June 2019 Human UniProt database (FCRL3 immunoprecipitation) or February 2024 (UBASH3A immunoprecipitations) and a common contaminants database (247 entries). Enzyme specificity was set as “Trypsin/P” with a maximum of two missed cleavages and minimum length of seven amino acids. N-terminal protein acetylation, methionine oxidation, and lysine biotinylation (226.0776 Da) were set as variable modifications, and cysteine carbamidomethylation was set as a fixed modification. In the case of the FCRL3 immunoprecipitation experiment, to transfer identifications across samples based on mass and normalized retention times, match between runs was enabled, using a matching time window of 0.7 min and an alignment time window of 20 min. Label-free protein quantification (LFQ) was performed with MaxLFQ (Cox et al., 2014) with a minimum required peptide ratio count of 1. Data analysis was performed using Perseus software v.1.6.2.3 (Tyanova et al., 2016). After log_2_ transformation of LFQ intensities, biological replicates were grouped, and proteins were filtered for a minimum of four valid values in at least one group. Missing data points were then replaced by imputation from a normal distribution with 0.3 width and 1.8 downshift, and a two-sided two-samples *t* test was used to identify significant changes of protein intensity between each immunoprecipitation experiment and its corresponding isotype control.

### Immunoprecipitation and western blots

HEK cells were transfected with plasmids to express FCRL3 and UBASH3A. After 48 h, cells were lysed in lysis buffer (25 mM Tris-HCl, pH 8.0, 250 mM NaCl, 0.2% NP-40, 0.5 mM EDTA, and 0.5 mM EGTA), supplemented with 1× proteinase inhibitor cocktail (Sigma-Aldrich) and 1× phosphatase inhibitor (Sigma-Aldrich) for 30 min on ice. The lysate was then cleared by centrifugation at 4°C at 13,000 × *g*. Protein concentration was determined using a BCA assay (Thermo Fisher Scientific, Pierce BCA Protein Assay Kit), and 700 µg of proteins was used for immunoprecipitation. To minimize nonspecific binding, the extracted protein was initially incubated with 10 μl of protein A magnetic beads (Thermo Fisher Scientific) for 1 h at 4°C using a rotator. Subsequently, the protein extract was incubated with 30 μl of protein A magnetic beads (previously washed with lysis buffer) and 1.5 µg of polyclonal rabbit anti-FCRL3 antibody (HPA048022-100UL; Sigma-Aldrich) overnight at 4°C in a total volume of 400 μl. The beads were then separated magnetically and washed twice with lysis buffer and twice with PBS. For western blot analysis, 50 μg of protein extract (input) or the entire immunoprecipitation was loaded on an 8% polyacrylamide gel after the addition of Laemmli buffer and boiling for 5 min. Blotting on a polyvinylidene difluoride membrane was performed using a methanol-based transfer buffer (20 mM Tris, 150 mM glycine, and 20% methanol). Blocking was performed with 5% milk in TBST (5 mM Tris, pH 7.3, 150 mM NaCl, and 0.1% Tween-20) for 60 min at room temperature with gentle shaking. Membranes were then incubated with primary antibodies at 4°C, followed by development with HRP-conjugated secondary antibodies. Analysis was performed with a Vilber Fusion FX blot imager.

### Data analysis

Statistical analysis was performed with Prism version 10 (GraphPad). Data visualization was performed using R (version 4.3.0). Venn diagrams were generated using Venny 2.0 (Oliveros, 2007).

### Online supplemental material

Fig. S1 provides additional characterization of human FCRL3^+^ T cells, Fig. S2 shows the association between the genotype of the donors and FCRL3 expression, Fig. S3 shows additional functional properties of FCRL3^+^ T cells, Fig. S4 shows data pertaining specifically to CD4^+^FCRL3^+^ T cells, both conventional and Tregs, Fig. S5 contains additional controls and mass-spec datasets for the FCRL3-UBASH3A interaction. Table S1 contains detailed information about the materials used in this study. Data S1 contains the RNA-seq datasets, Data S2 and S3 contain all mass-spec datasets, and Data S4 provides data underlying the figures in this study.

## Supplementary Material

Table S1 shows materials used in this study.

Data S1 shows RNA-seq results from human CD4^+^, CD8^+^, and Treg T cells, FCRL3^+^ versus FCRL3^−^ cells.

Data S2 shows shotgun proteomics of CD8^+^ T cells, FCRL3^+^ versus FCRL3^−^.

Data S3 shows TurboID-MS and IP and mass spectrometry datasets.

Data S4 provides data underlying the figures in this study.

SourceData F5 is the source file for Fig. 5.

SourceData F6 is the source file for Fig. 6.

SourceData FS2 is the source file for Fig. S2.

SourceData FS5 is the source file for Fig. S5.

## Data Availability

Materials are available in Addgene (https://www.addgene.org/Silvia_Monticelli/) or upon request. The Protein A-TurboID construct belongs to Michiel Vermeulen. All data are available in the main text, in the supplementary materials, or in GEO with the accession number GSE241004. TCR Vβ sequences are shared through the immuneACCESS data portal (https://clients.adaptivebiotech.com/pub/bianchi-2025-jem).
