# Supplementary material for: FCRL3 is an immunoregulatory receptor that restrains the activation of human memory T lymphocytes
Source: J Exp Med. 2025 Oct 15;223(1):e20242474. doi: 10.1084/jem.20242474 (PMC12524113; doi:10.1084/jem.20242474)
Supplement: Table S1 — shows materials used in this study. [file jem_20242474_tables1.docx]

***Table S1. Materials used in this study***

| **REAGENT** | **SOURCE** | **IDENTIFIER** |
| --- | --- | --- |
| Antibodies | | |
| Bispecific monoclonal antibody against human CD19 and human CD3 | InvivoGen | bimab-hcd19cd3. Clone HD37 (Anti-hCD19) and L2K-07 (Anti-hCD3) |
| Mouse monoclonal anti-human CD307c/FCRL3 PE | BioLegend | Cat#374406; clone H5 |
| Mouse monoclonal anti-human CD307c/FCRL3 PE/Cyanine7 | BioLegend | Cat#374409; clone H5 |
| Mouse monoclonal anti-human CD8 FITC | BioLegend | Cat#344703; clone SK1 |
| Mouse monoclonal anti-human CD197 (CCR7) Brilliant Violet 421 | BioLegend | Cat#353208; clone G043H7 |
| Mouse monoclonal anti-human CD196 (CCR6) Brilliant Violet 605 | BioLegend | Cat#353419; clone G034E3 |
| Mouse monoclonal anti-human CD183 (CXCR3) Alexa Fluor® 647 | BioLegend | Cat#353711; clone G025H7 |
| Mouse monoclonal anti-human CD25 PE | BioLegend | Cat#356103; clone M-A251 |
| Mouse monoclonal anti-human CD279 (PD-1) | BioLegend | Cat#329951; clone EH12.2H7 |
| Mouse monoclonal anti-human IFN-g APC/Cyanine7 | BioLegend | Cat#502530; clone 4S.B3 |
| Mouse monoclonal anti-human Granzyme B Alexa Fluor® 647 | BioLegend | Cat#515405; clone GB11 |
| Mouse monoclonal anti-human Perforin PE/Cyanine7 | BioLegend | Cat#353315; clone B-D48 |
| Mouse monoclonal anti-human CD4 PE-Texas Red | Invitrogen, ThermoFisher Scientific | Cat#MHCD0417; clone S3.5 |
| Mouse monoclonal anti-human CD45RA Qdot 655 Conjugate | Invitrogen, ThermoFisher Scientific | Cat#Q10069; clone MEM-56 |
| Monoclonal anti-human CD25 PC5 | Beckman Coulter | Cat#IM2646U; clone B1.49.9 |
| Brilliant Violet 421 anti-human CD25 Antibody | BioLegend | Cat#356113; clone  M-A251 |
| Mouse monoclonal Anti-human CD107a BV421 | BD Horizon | Cat#562623; clone H4A3 |
| Mouse monoclonal anti-human CD8 BV786 | BD Horizon | Cat#563823; clone RPA-T8 |
| Rat monoclonal anti-human TOX RB780 | BD Horizon | Cat#570193; clone NAN448B |
| Mouse monoclonal anti-human EOMES, FITC | ThermoFisher | Cat#11-4877-42; clone  WD1928 |
| Mouse monoclonal anti-human Ox40, BV421 | BioLegend | Cat#350013; clone ACT35 |
| Mouse monoclonal anti-human Ox40, FITC | BioLegend | Cat#350006; clone ACT35 |
| Mouse monoclonal anti-human CD25, FITC | BioLegend | Cat#356105; clone M-A251 |
| Mouse monoclonal anti-human CD137, APC | BioLegend | Cat#309809; clone 4B4-1 |
| Mouse monoclonal anti-human CD40L, APC/Cyanine7 | BioLegend | Cat#310822; clone 24-31 |
| Mouse monoclonal anti-human CD28 | BD Biosciences | Cat#555725; clone CD28.2 |
| Mouse monoclonal anti-human CD194 (CCR4) PE/Cyanine7 | BD Biosciences | Cat#557864; clone 1G1 |
| Mouse monoclonal anti-human CD3 | Recombinant, prepared in house | Clone TR66 |
| Ultra-LEAF purified anti-human CD307c/FCRL3 Antibody | BioLegend | Cat#374403; clone H5 |
| Anti-human EGFR Antibody, PE | BioLegend | Cat#352903; clone AY13 |
| EGFR Antibody (D-8) | Santa Cruz | Cat#sc-365829 |
| Anti-human UBASH3A-Specific Polyclonal antibody | Proteintech | Cat#15823-1-AP |
| Goat anti-Rabbit IgG (H+L) Cross-Adsorbed Secondary Antibody, Alexa Fluor 750 | Invitrogen, ThermoFisher Scientific | Cat#A-21039 |
| Anti-human CD3ε-FITC antibody | Beckman Coulter | Cat#A07746 |
| Anti-DYKDDDDK Tag Antibody, BV421 | BioLegend | Cat# 637321 |
| Chemicals and recombinant cytokines | | |
| Human IL-2 | Recombinant, prepared in house |  |
| Recombinant human IL-15 | Peprotech | Cat#200-15-10UG |
| Recombinant human IL-7 | Peprotech | Cat#200-07-10UG |
| True Cut Cas9 Protein v2 | ThermoFisher Scientific | Cat#A36499 |
| Poly-L-glutamic acid sodium salt (PGA) | Sigma | Cat#P4761-25MG |
| Alt-R CRISPR-Cas9 tracRNA | IDT | Cat#1072532 |
| Alt-R CRISPR-Cas9 Electroporation Enhancer | IDT | Cat#1075915 |
| LIVE/DEAD Fixable Blue Dead Cell Stain Kit, for UV excitation | ThermoFisher Scientific | Cat#L23105 |
| LIVE/DEAD Fixable Aqua Dead Cell Stain Kit | ThermoFisher Scientific | Cat#L34957 |
| KOD Hot Start DNA Polymerase | Sigma | Cat#71086 |
| PMA | Sigma | Cat#P1585-1MG |
| Ionomycin calcium salt | Sigma | Cat#I0634-1MG |
| Brefeldin A | Sigma | Cat#B6542-5MG |
| PhosSTOP | Sigma | Cat#4906845001 |
| Protease Inhibitor cocktail | Sigma | Cat#P8340-1ML |
| BD GolgiStop Protein Transport Inhibitor (Containing Monensin) | BD biosciences | Cat#554724 |
| SYBR Green FastMix | Quanta Bioscience/VWR | Cat#733-1389 |
| qScript cDNA SuperMix | Quanta Bioscience/VWR | Cat#733-1177 |
| Lipofectamine 3000 Transfection Reagent | Invitrogen | Cat#L3000008 |
| In-Fusion Snap Assembly Master Mix | Takara Bio | Cat# 638947 |
| IntraSure Kit | BD Biosciences | Cat# 641778 |
| E.Z.N.A. Plasmid DNA Mini Kit I | Omega Bio-Tek | Cat#D6943-02 |
|  |  |  |
| Key commercial assays | | |
| APC BrdU Kit | BD Biosciences | Cat#552598 |
| QIAamp DNA Micro Kit | Qiagen | Cat#56304 |
| P3 Primary Cell 4D-Nucleofector X Kit S | Lonza Bioscience | Cat#LZ-V4XP-3032 |
| QuikChange II XL Site-Directed Mutagenesis Kit | Agilent | Cat#: 200521 |
| Deposited data | | |
| RNA-seq data | This paper | GEO: GSE123465 |
| TCR sequencing | This paper | Adaptive Biotechnologies |
| Cell lines | | |
| Human: JeKo-1 | ATCC | CRL-3006 |
| Human: HEK-293 | ATCC | CRL-1573 |
| Human: Jurkat | ATCC | TIB-152 |
| Oligonucleotides | | |
| Primers for *FCRL3* genotyping, SNP “C” Forward 5’- GGGTGAGATTACGGGAAGTCCC-3’ | This paper | N/A |
| Primers for *FCRL3* genotyping, SNP “T” Forward 5’- GGGTGAGATTACGGGAAGTCCT-3’ | This paper | N/A |
| Primers for *FCRL3* genotyping, Reverse 5’-TCACACAGCCTTTGGTTCTG-3’ | This paper | N/A |
| Primer for pLVX IRES-ZsGreen-Blpl mutagenesis, Forward 5’- GTTTTCCTTTGAAAAACACGATGATAAGCTTAGCTATGGCCCAGTCCAA-3’ | This paper | N/A |
| Primer for pLVX IRES-ZsGreen-BlpI mutagenesis, Reverse 5’- TTGGACTGGGCCATAGCTAAGCTTATCATCGTG TTTTTCAAAGGAAAAC-3’ | This paper | N/A |
| Primer for pLVX IRES-ZsGreen- AsiSI mutagenesis, Forward 5’-CCTTGCCCTGAACGCGATCGCTCTGGAACAATC AACC-3’ | This paper | N/A |
| Primer for pLVX IRES-ZsGreen- AsiSI mutagenesis, Reverse 5’-GGTTGATTGTTCCAGAGCGATCGCGTTCAGGGC AAGG-3’ | This paper | N/A |
| Primers for PuroR cloning into pLVX backbone; BlpI restriction site; Forward 5’- AAAAGCTTAGCATGACCGAGTACAAGCCCAC-3’ | This paper | N/A |
| Primers for PuroR cloning into pLVX backbone; AsiSI restriction site; Reverse 5’-AAAAACTAGTGCCACCATGCTTCTGTGGCTGCT GCTGCTG-3’ | This paper | N/A |
| Primers for *FCRL3* cloning into pLVX Forward 5’-AAAAACTAGTGCCACCATGCTTCTGTGGCTGCT GCTGCTG-3’ | This paper | N/A |
| Primers for *FCRL3* cloning into pLVX Reverse 5’-AAAAGCGGCCGCCTAGTGGTCTGAGGCCAGTAATACA-3’ | This paper | N/A |
| Primers for cloning FCLR3-EGFR chimera into pLVX; EGFR Extracellular Domain amplification, Forward 5’-ATGCGACCCTCCGGGA-3’ | This paper | N/A |
| Primers for cloning FCLR3-EGFR chimera into pLVX; EGFR Extracellular domain amplification, Reverse 5’ GGACGGGATCTTAGGCCCA | This paper | N/A |
| Primers for cloning FCLR3-EGFR chimera into pLVX; FCRL3 Transmembrane domain + Cytoplasmic Tail+ overlapping 5`end; Forward 5’-CCTAAGATCCCGTCCGCTGCGGGAATCACG-3’ | This paper | N/A |
| Primers for cloning FcLR3-EGFR chimera into pLVX; FCRL3 transmembrane-intracellular domains amplification; Reverse 5’-CTAGTGGTCTGAGGCCAGTAATACAC-3’ | This paper | N/A |
| Primers for cloning FCLR3-EGFR chimera into pLVX; Full chimera amplification + restriction site XbaI; Forward; 5’ AAAATCTAGAATGCGACCCTCCGGGACG 3’ | This paper | N/A |
| Primers for cloning FCLR3-EGFR chimera into pLVX; Full chimera amplification + restriction site NotI Reverse; 5’ AAAAGCGGCCGCCTAGTGGTCTGAGGCCA 3’ | This paper | N/A |
| Primers for cloning Chimera_93aa_Rev; Chimera amplification + NotI restriction site; 5’-AAAAGCGGCCGCCTATTCCTCATGCTCTTG -3’ | This paper | N/A |
| Primers for cloning Chimera_50aa_Rev; Chimera amplification + NotI restriction site; 5’- AAAAGCGGCCGCCTACATTGGGGCTAGTGG -3’ | This paper | N/A |
| Sybr green RT-qPCR; *UBE2D2* FW  5’-GATCACAGTGGTCTCCAGCA-3’ | This paper | N/A |
| Sybr green RT-qPCR; *UBE2D2* REV  5’-CGAGCAATCTCAGGCACTAA-3’ | This paper | N/A |
| Sybr green RT-qPCR; *EOMES* FW  5’-AGGCGCATGTTTCCTTTCT-3’ | This paper | N/A |
| Sybr green RT-qPCR; *EOMES* REV  5’-GCACCACCTCTACGAACACA-3’ | This paper | N/A |
| Sybr green RT-qPCR; *TOX* FW  5’-GCTACCATTTCATCCACAAAA-3’ | This paper | N/A |
| Sybr green RT-qPCR; *TOX* REV  5’-CTGAGGATGGGAACTGTACTGA-3’ | This paper | N/A |
| Sybr green RT-qPCR; *FCRL3* FW  5’-CCAGCACAGTCATGGAGTGA-3’ | This paper | N/A |
| Sybr green RT-qPCR; *FCRL3* REV  5’-CAGTACAGGATCGGGAAGGA-3’ | This paper | N/A |
| Sybr green RT-qPCR; *FOXP3* FW  5’-CAAATGGTGTCTGCAAGTGG-3’ | This paper | N/A |
| Sybr green RT-qPCR; *FOXP3* REV  5’-TGCCCTTCTCATCCAGAAGAT-3’ | This paper | N/A |
| sgRNA 1, targeting exon 4 of *FCRL3*: 5’- AATTACCAATGTAAGACCCG | This paper | N/A |
| sgRNA 2, targeting exon 7 of *FCRL3*: 5’- AATCTAGAGATCCGGCCCAC | This paper | N/A |
| Control sgRNA: 5’- GGTTCTTGACTACCGTAATT | This paper | N/A |
| Primer for cloning 3xFLAG into pLVX-EF1α-FCRL3-IRES-puro with In-Fusion Cloning; Forward 5’-GCGGCCGCGGATCC-3’ | This paper | N/A |
| Primer for cloning 3xFLAG into pLVX-EF1α-FCRL3-IRES-puro with In-Fusion Cloning; Reverse 5’GTGGTCTGAGGCCAGTAATACACG-3’ | This paper | N/A |
| Primer for cloning 3xFLAG into pLVX-EF1α-FCRL3-IRES-puro with In-Fusion Cloning; Reverse 5’GGGATCCGCGGCCGCGTTTAAACCTTATCGT-3’ | This paper | N/A |
| Primer for cloning 3xFLAG into pLVX-EF1α-FCRL3-IRES-puro with In-Fusion Cloning; Forward 5’CTGGCCTCAGACCACCGGCCGCTCGAGGATTAC-3’ | This paper | N/A |
| Primers for cloning FCRL3-93aa into pLVX-EF1α-FCRL3-puro+ScaI restriction site Forward:  5’GTGTCTTCTTCAGTTCTGAATAGAGTACTGTTCATTCCTCATGCTCTTGATGCATCATTG- ‘3 | This paper | N/A |
| Primers for cloning FCRL3-93aa into pLVX-EF1α-FCRL3-puro+ScaI restriction site Reverse:  5’CAATGATGCATCAAGAGCATGAGGAATGAACAGTACTCTATTCAGAACTGAAGAAGACAC- ‘3 | This paper | N/A |
| Primers for cloning FCRL3-50aa into pLVX-EF1α-FCRL3-puro+XhoI restriction site Forward:  5’-ACA TTG CTG TAC ATT GGC TCG AGT TAC ATT GGG GCT AGT GGT TTA G-‘3 | This paper | N/A |
| Primers for cloning FCRL3-50aa into pLVX-EF1α-FCRL3-puro+XhoI restriction site Reverse:  5’-CTA AAC CAC TAG CCC CAA TGT AAC TCG AGC CAA TGT ACA GCA ATG T-‘3 | This paper | N/A |
| Primers for cloning FCRL3-16aa into pLVX-EF1α-FCRL3-puro+AgeI restriction site Forward:  5’-CAC TAG GAC TGT GAC TAG ATT AAC CGG TGG CAG AAA GTC CTC CTG G- ‘3 | This paper | N/A |
| Primers for cloning FCRL3-16aa into pLVX-EF1α-FCRL3-puro+AgeI restriction site Reverse:  5’-CCA GGA GGA CTT TCT GCC ACC GGT TAA TCT AGT CAC AGT CCT AGT G- ‘3 | This paper | N/A |
| Plasmids | | |
| pLVX-EF1α-FCRL3-IRES-puro | This paper | N/A |
| pLVX-EF1α-IRES-puro | This paper | N/A |
| pLVX-EF1α-EGFR-FCRL3-50aa-Chimera-puro | This paper | N/A |
| pLVX-EF1α-EGFR- FCRL3-93aa-Chimera-puro | This paper | N/A |
| pLVX-EF1α-EGFR-FCRL3-140aa-Chimera-puro | This paper | N/A |
| pLVX-EF1α-FCRL3-16aa-puro | This paper | N/A |
| pLVX-EF1α-FCRL3-50aa-puro | This paper | N/A |
| pLVX-EF1α-FCRL3-93aa-puro | This paper | N/A |
| pLVX-EF1α-FCRL3-3xFLAG-puro | This paper | N/A |
| ProtA-TurboID | Santos-Barriopedro et al., 2021 |  |
| Other | | |
| *Escherichia coli* strain C3013 | NEB | Cat#C3013I |
| LB-agar | Sigma | Cat#L2897 |
| LB medium | Sigma | Cat#L3032 |
| IPTG | Sigma | Cat#I6758 |
| streptavidin beads | Sigma | Cat# S1638-1ML |
| Ni-NTA agarose beads | Qiagen | Cat#30210 |
| DYKDDDDK Fab-Trap Agarose | ChromoTek | Cat#ffa-10 |
| Pierce Disposable Column | Thermo Scientific | Cat# 29924 |
| Imperial Protein Staining | Thermo Scientific | Cat# 24615 |
| MWCO spin concentrator | Millipore | Cat#UFC901008 |
| 0.22 μm Millex-GP filter | Millipore | Cat#SLMP025SS |
| Instruments |  |  |
| Fiberlite F9-6 x 1000 | Thermo Scientific | Centrifuge |
| Fiberlite F20-12 x 50 LEX | Thermo Scientific | Centrifuge |
