# Supplementary figures and images for: FCRL3 is an immunoregulatory receptor that restrains the activation of human memory T lymphocytes
Source: J Exp Med. 2025 Oct 15;223(1):e20242474. doi: 10.1084/jem.20242474 (PMC12524113; doi:10.1084/jem.20242474)

SourceDataF5F

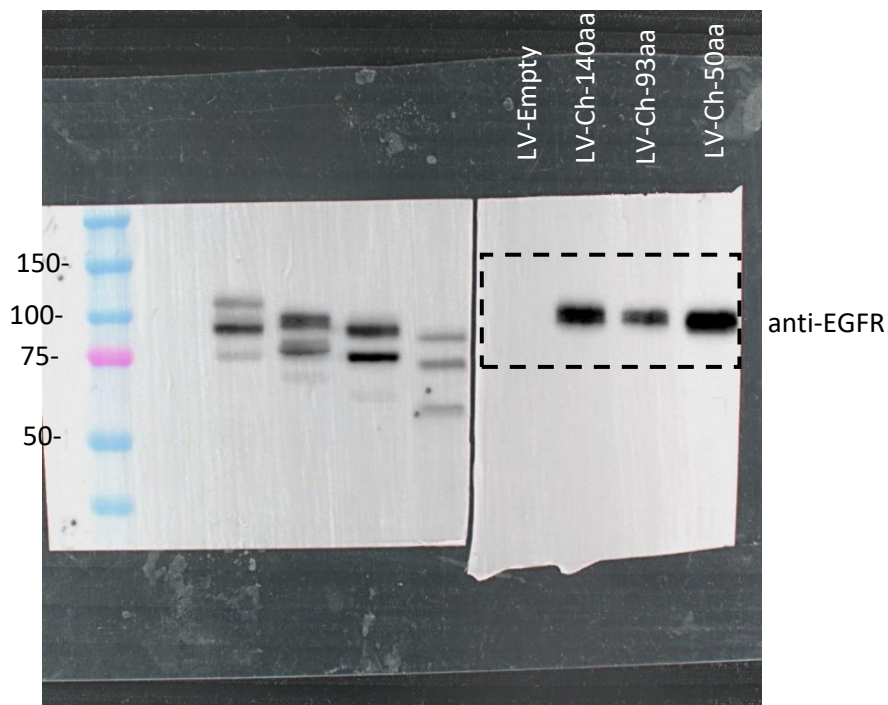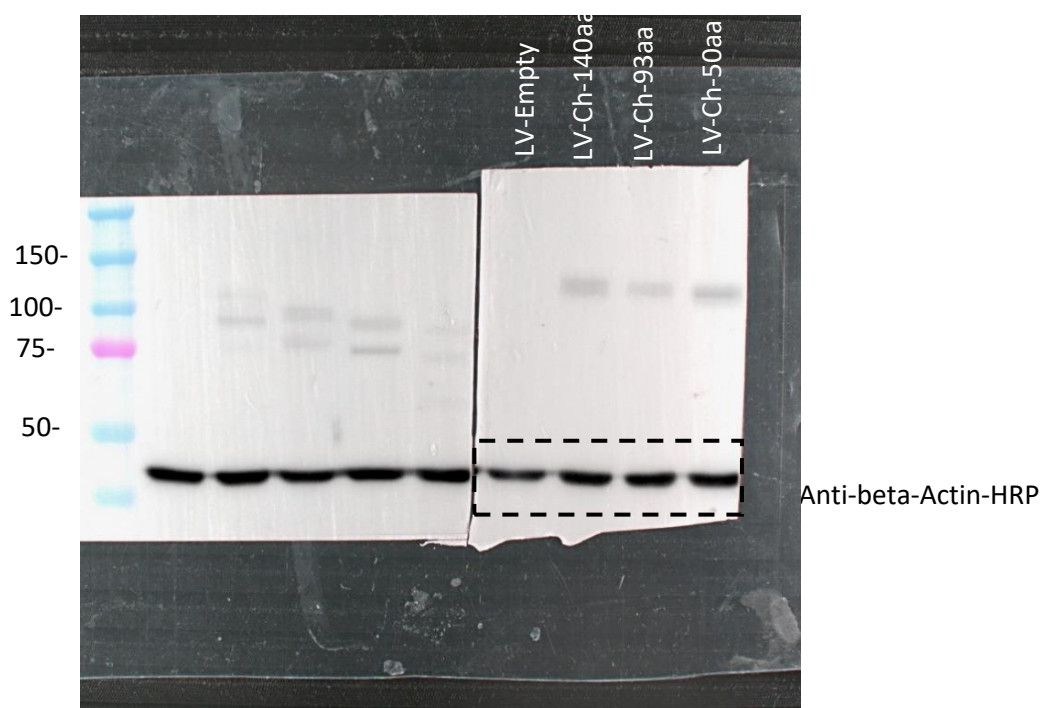

Supplement: SourceData F5 — is the source file for Fig. 5. [file jem_20242474_sourcedataf5.pdf]

SourceDataF6B

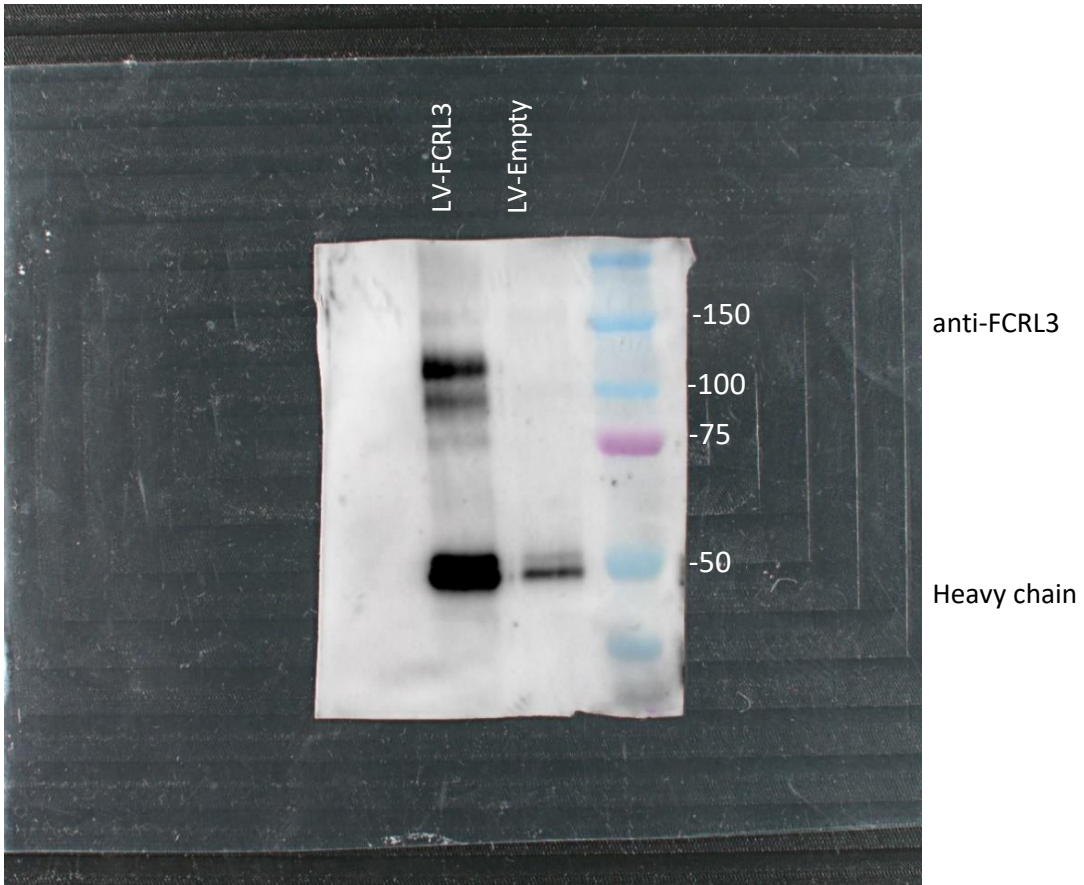

SourceDataF6D

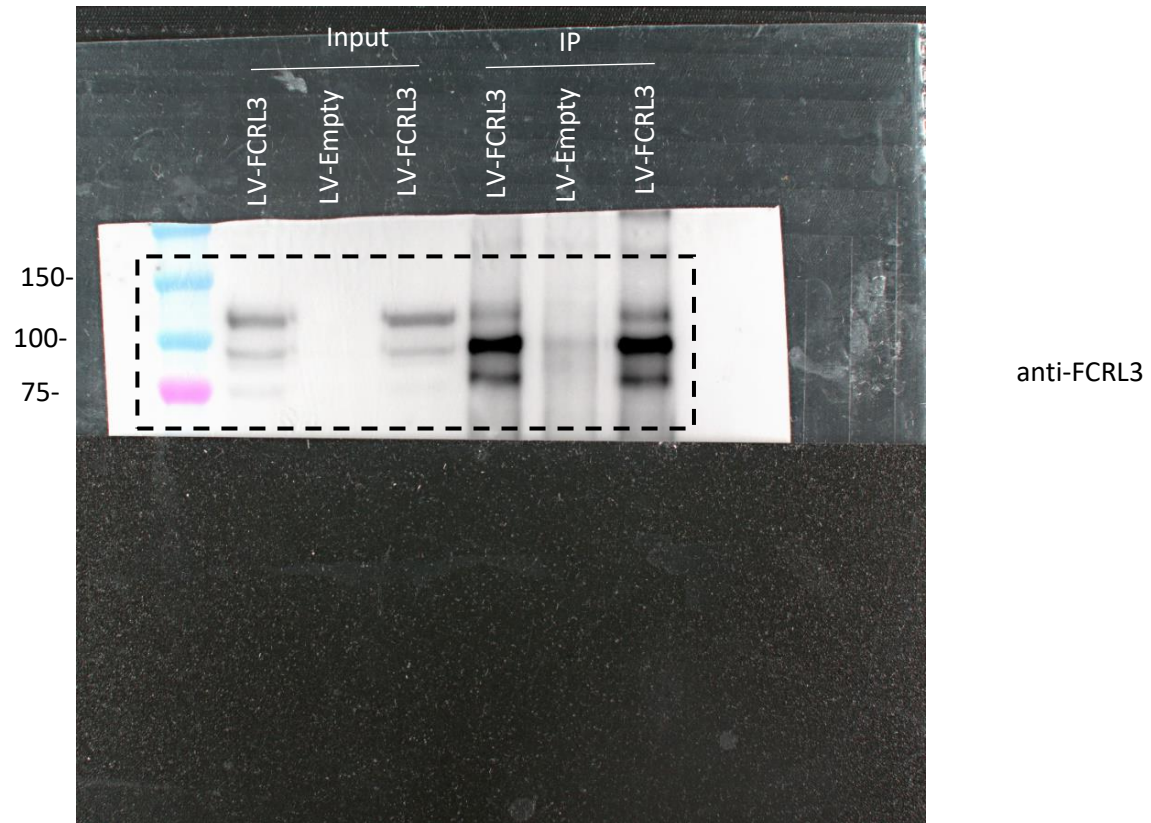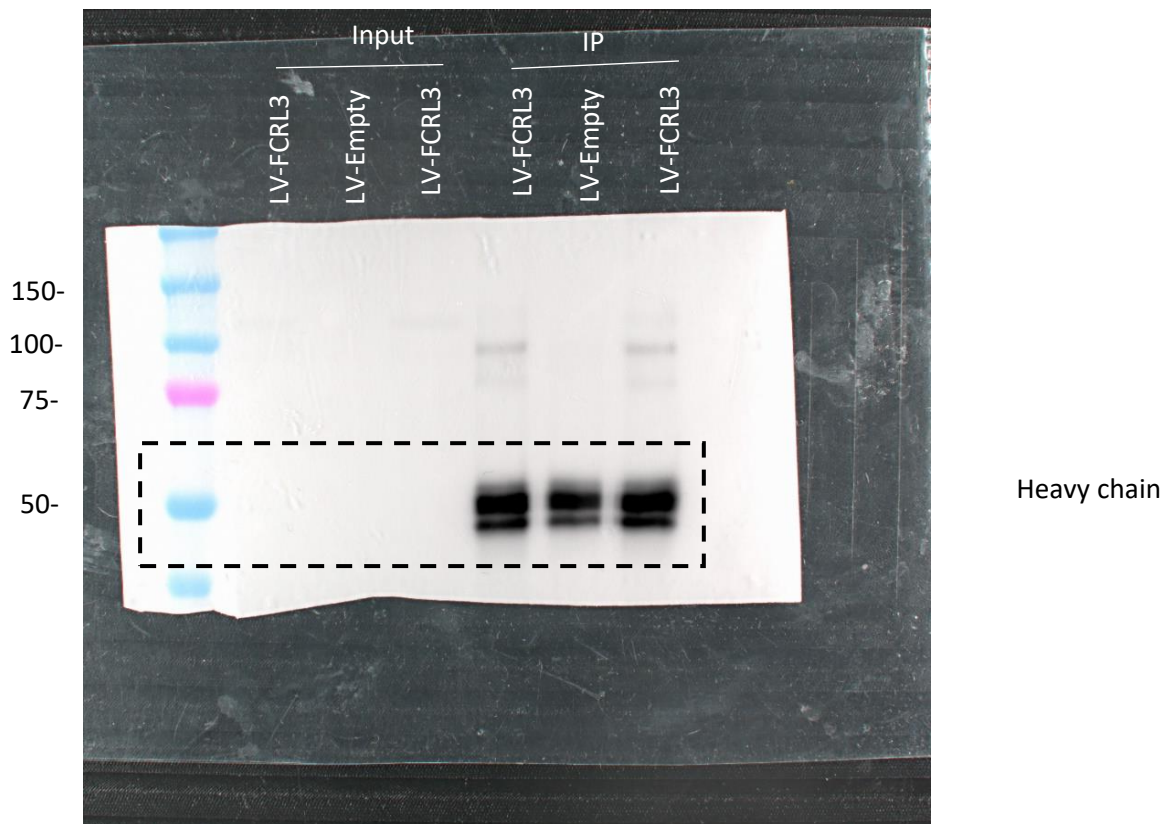

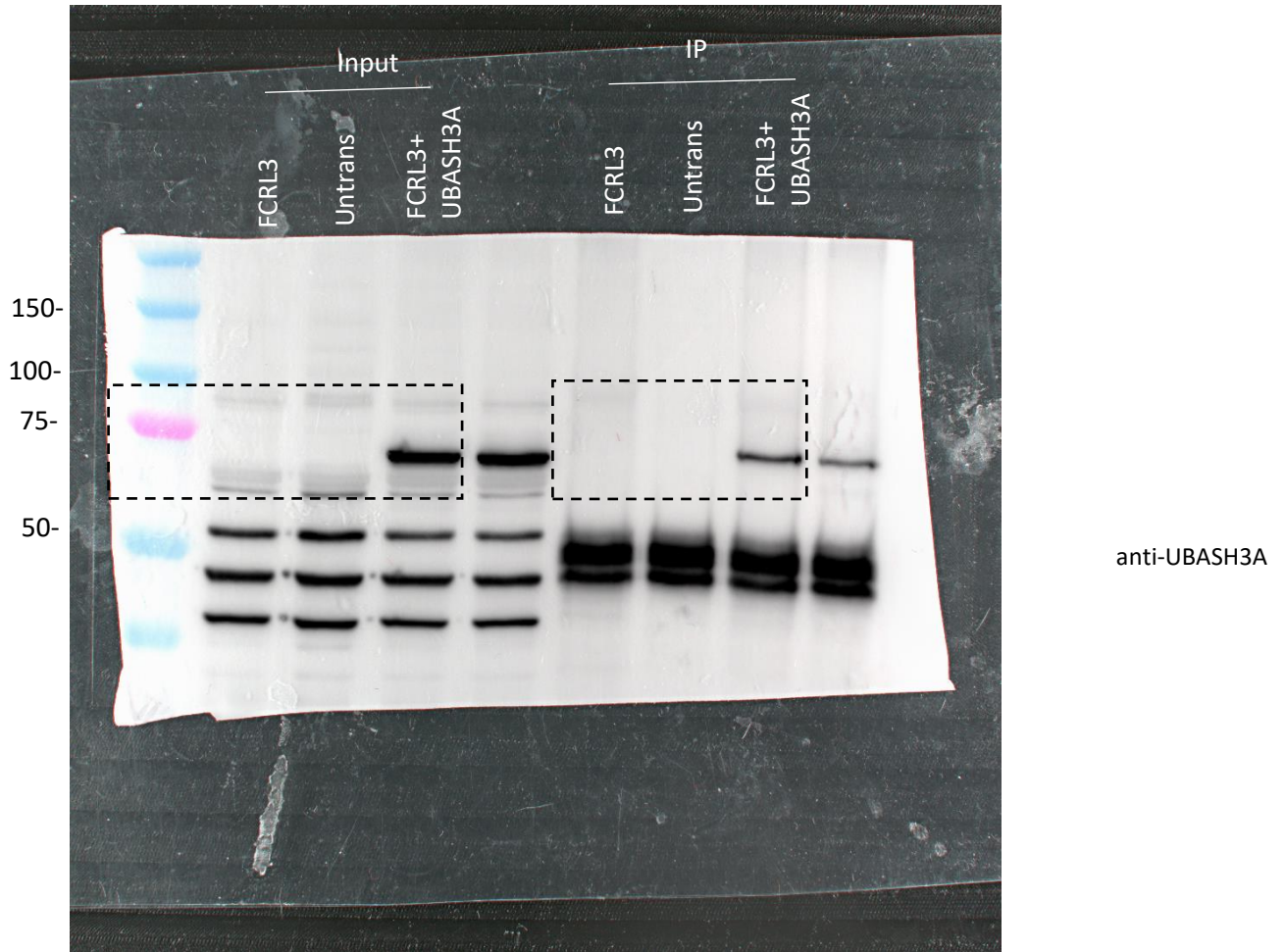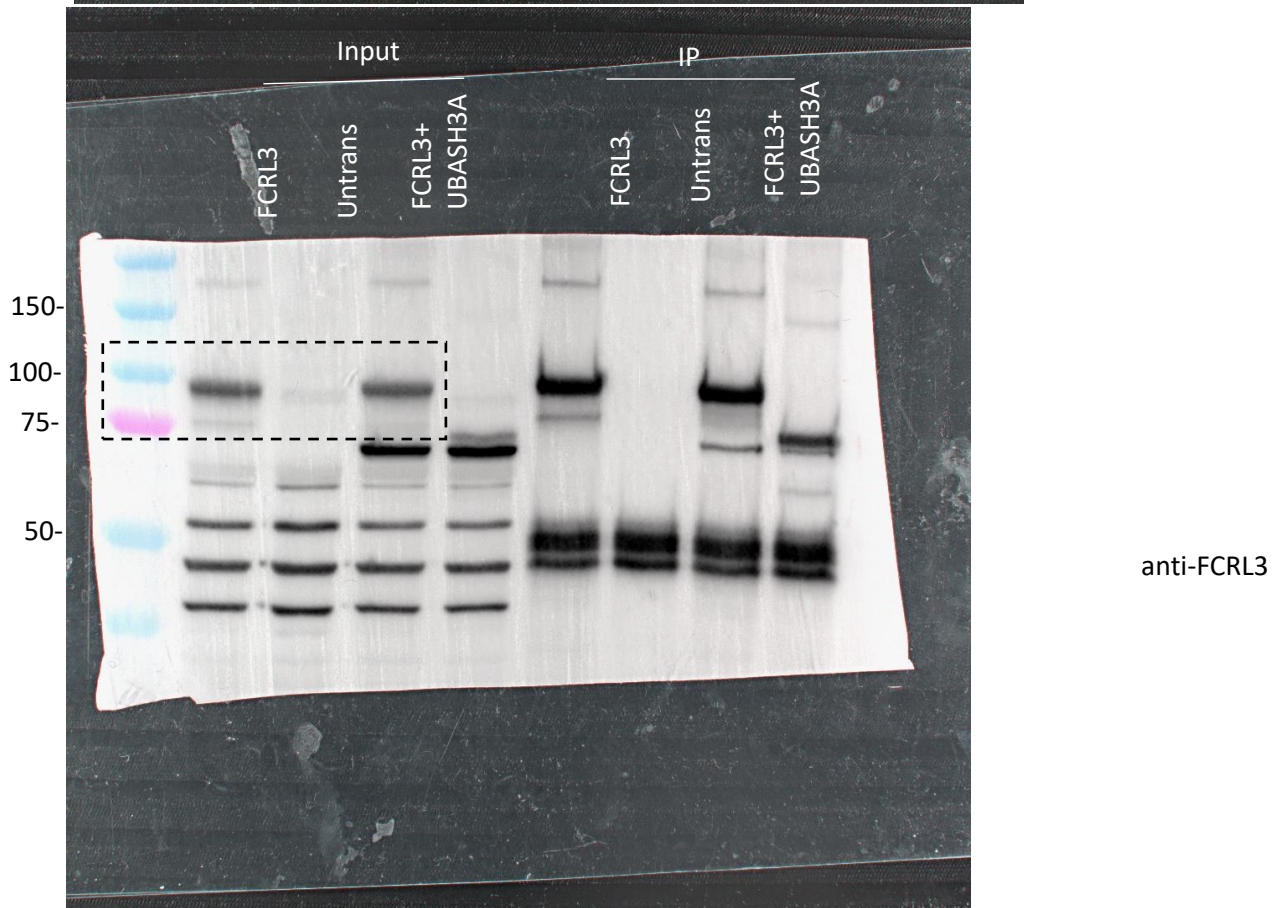

SourceDataF6G

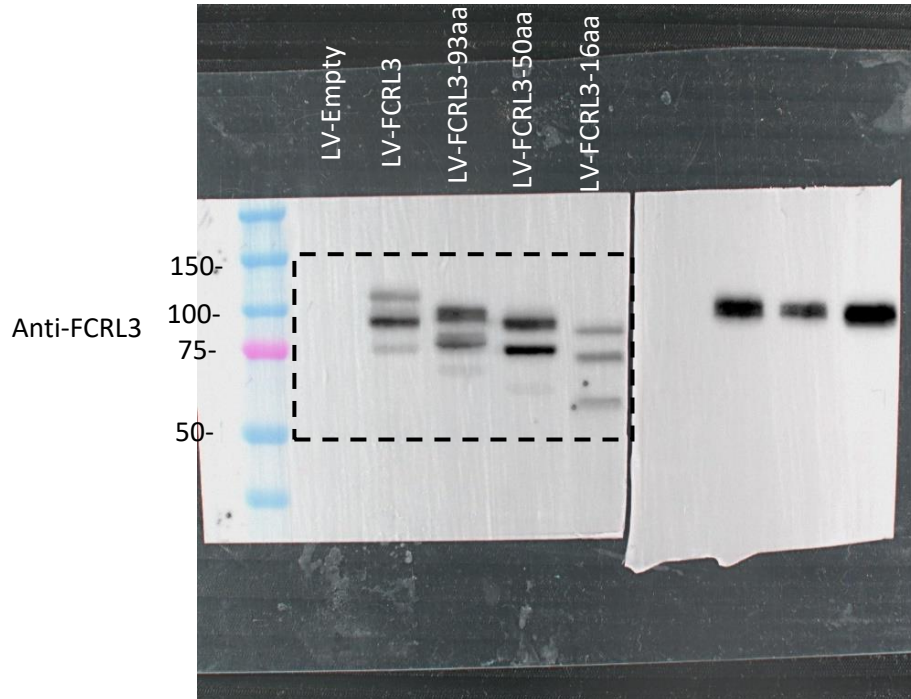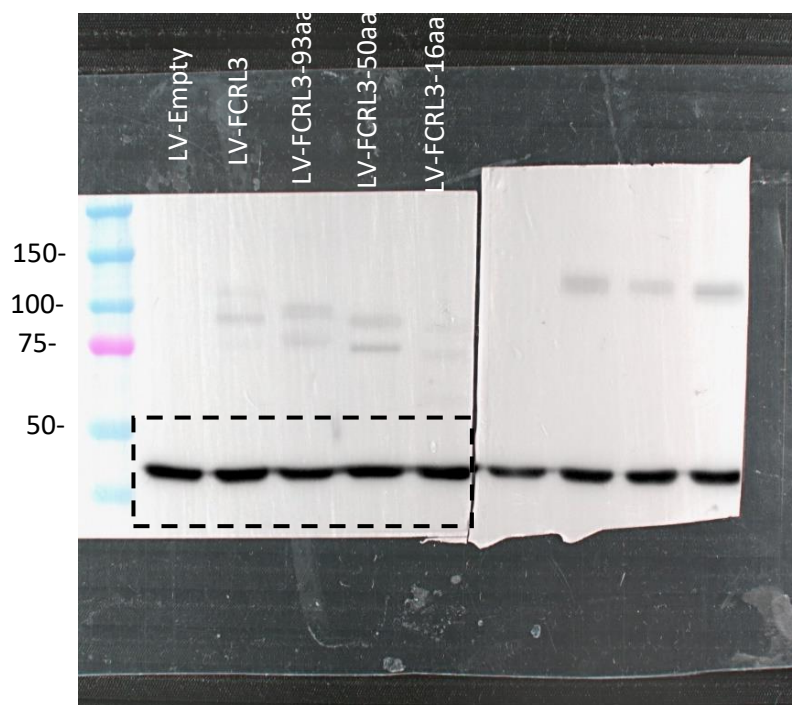

Anti-beta-Actin-HRP

Supplement: SourceData F6 — is the source file for Fig. 6. [file jem_20242474_sourcedataf6.pdf]

SourceDataFS2C

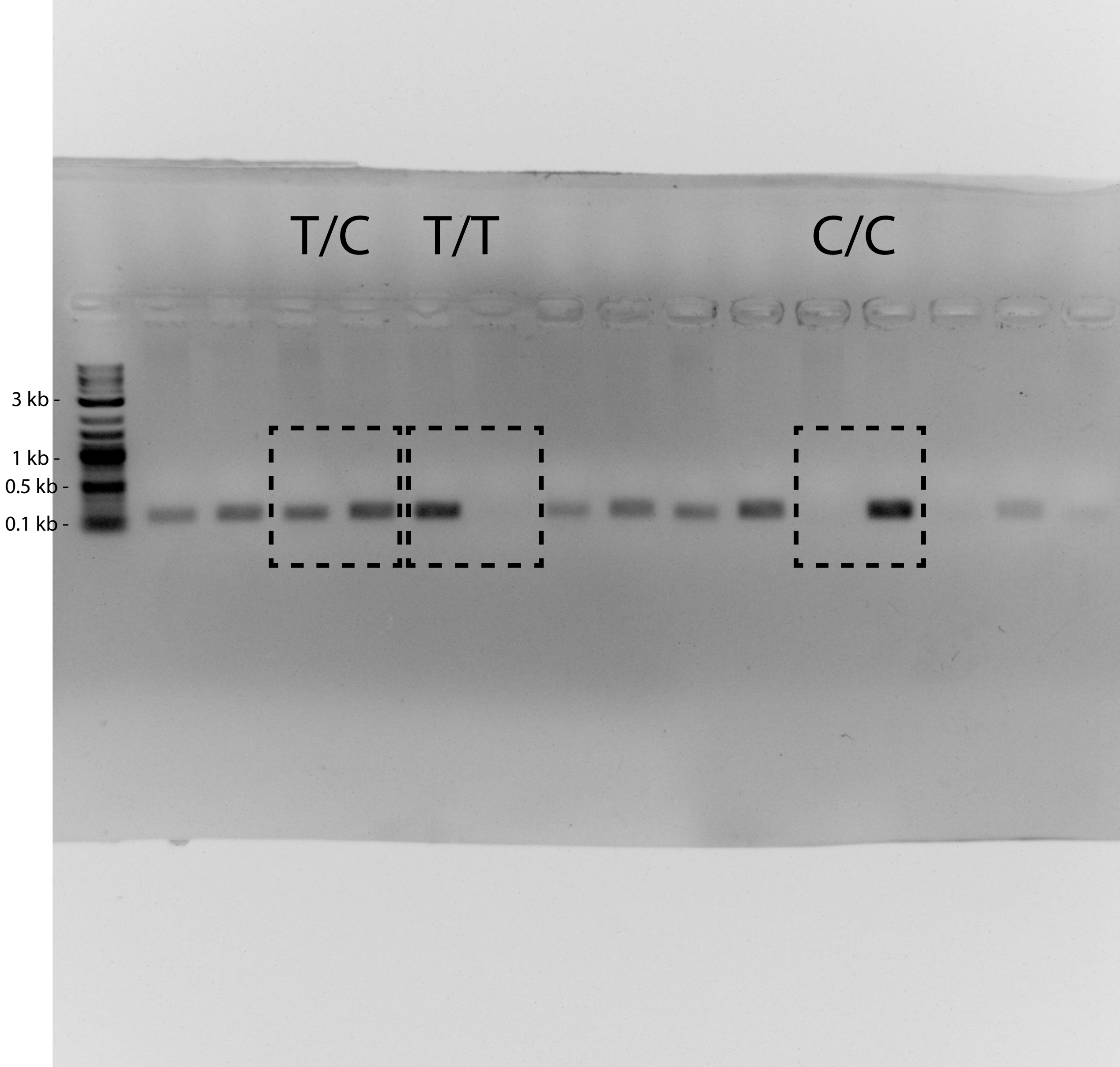

Supplement: SourceData FS2 — is the source file for Fig. S2. [file jem_20242474_sourcedatafs2.pdf]

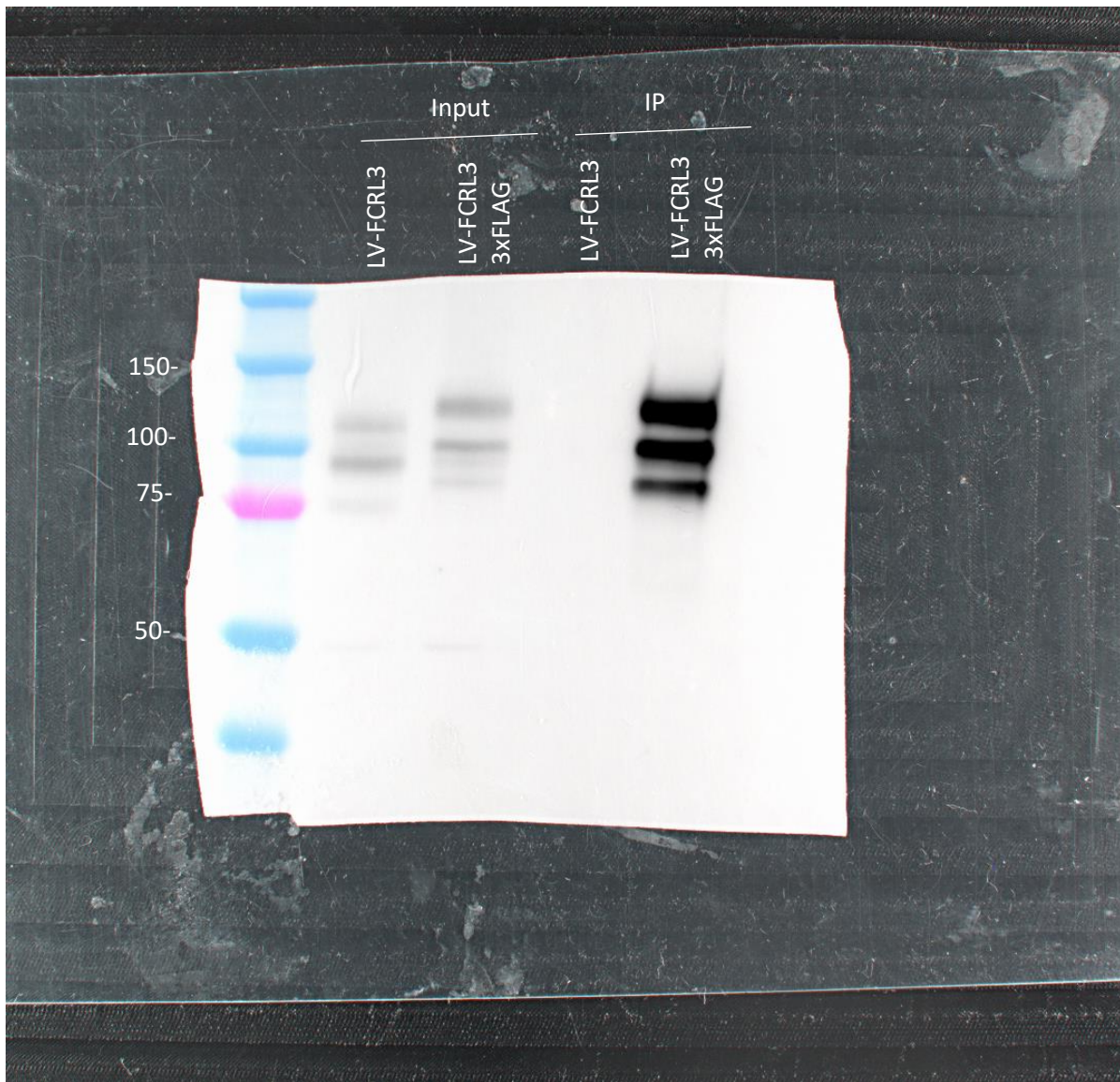

IB:FCRL3

Supplement: SourceData FS5 — is the source file for Fig. S5. [file jem_20242474_sourcedatafs5.pdf]
